# Interleukin-22 Deficiency Contributes to Dextran Sulfate Sodium-Induced Inflammation in Japanese Medaka, *Oryzias latipes*

**DOI:** 10.3389/fimmu.2021.688036

**Published:** 2021-10-25

**Authors:** Yoshie Takahashi, Yo Okamura, Nanaki Harada, Mika Watanabe, Hiroshi Miyanishi, Tomoya Kono, Masahiro Sakai, Jun-ichi Hikima

**Affiliations:** ^1^ International Course of Agriculture, Graduate School of Agriculture, University of Miyazaki, Miyazaki, Japan; ^2^ Interdisciplinary Graduate School of Agriculture and Engineering, University of Miyazaki, Miyazaki, Japan; ^3^ Department of Biochemistry and Applied Biosciences, Faculty of Agriculture, University of Miyazaki, Miyazaki, Japan; ^4^ Department of Marine Biology and Environmental Sciences, Faculty of Agriculture, University of Miyazaki, Miyazaki, Japan

**Keywords:** transcriptome analysis, interleukin-22 receptor, interleukin-22, inflammation, genome editing, Japanese medaka

## Abstract

Mucosal tissue forms the first line of defense against pathogenic microorganisms. Cellular damage in the mucosal epithelium may induce the interleukin (IL)-22-related activation of many immune cells, which are essential for maintaining the mucosal epithelial barrier. A previous study on mucosal immunity elucidated that mammalian IL-22 contributes to mucus and antimicrobial peptides (AMPs) production and anti-apoptotic function. IL-22 has been identified in several teleost species and is also induced in response to bacterial infections. However, the roles of IL-22 in teleost immunity and mucus homeostasis are poorly understood. In this study, Japanese medaka (*Oryzias latipes*) was used as a model fish. The medaka *il22*, il22 receptor A1 (*il22ra1*), and il22 binding protein (*il22bp*) were cloned and characterized. The expression of medaka *il22*, *il22ra1*, and *il22bp* in various tissues was measured using qPCR. These genes were expressed at high levels in the mucosal tissues of the intestines, gills, and skin. The localization of *il22* and *il22bp* mRNA in the gills and intestines was confirmed by *in situ* hybridizations. Herein, we established IL-22-knockout (KO) medaka using the CRISPR/Cas9 system. In the IL-22-KO medaka, a 4-bp deletion caused a frameshift in *il22*. To investigate the genes subject to IL-22-dependent regulation, we compared the transcripts of larval medaka between wild-type (WT) and IL-22-KO medaka using RNA-seq and qPCR analyses. The comparison was performed not only in the naïve state but also in the dextran sulfate sodium (DSS)-exposed state. At the transcriptional level, 368 genes, including immune genes, such as those encoding AMPs and cytokines, were significantly downregulated in IL-22-KO medaka compared that in WT medaka in naïve states. Gene ontology analysis revealed that upon DSS stimulation, genes associated with cell death, acute inflammatory response, cell proliferation, and others were upregulated in WT medaka. Furthermore, in DSS-stimulated IL-22-KO medaka, wound healing was delayed, the number of apoptotic cells increased, and the number of goblet cells in the intestinal epithelium decreased. These results suggested that in medaka, IL-22 is important for maintaining intestinal homeostasis, and the disruption of the IL-22 pathway is associated with the exacerbation of inflammatory pathology, as observed for mammalian IL-22.

## Introduction

Mucosal tissue forms the first line of defense against pathogenic microorganisms. Symbiotic microorganisms colonize the mucus layer, and their mutualistic relationships are vital to host health (1). Mucins secreted by goblet cells form a thick internal mucus layer (2). Antimicrobial peptides (AMPs) synthesized from epithelial Paneth cells and keratinocytes kill microbes or inhibit their growth (3). Physicochemical defenses help maintain mucosal homeostasis, and their dysfunction may induce various autoimmune diseases (4, 5).

In the mammalian mucosa, interleukin (IL)-22 is a key cytokine that maintains the epithelial barrier. It has attracted the attention of researchers as it is associated with skin inflammation and inflammatory bowel disease (IBD) (4, 6). IL-22 was first cloned from IL-9-stimulated murine T cells and characterized as an IL-10-related, T cell-derived inducible factor because it showed high amino acid (aa) sequence homology with IL-10 (7). Mammalian IL-22 belongs to the IL-10 cytokine family, which includes IL-10, IL-19, IL-20, IL-24, and IL-26. IL-22 is primarily produced by type 3 innate lymphoid, natural killer, T helper type (Th)-1, Th-17, and Th-22 cells as well as by neutrophils (8–10). IL-22 is synthesized and secreted in response to proinflammatory cytokines such as IL-1β, IL-6, TNF-α, and IL-23 (11). The activation of the aryl hydrocarbon receptor transcription factor promotes IL-22 synthesis in immunocytes that secrete it (12). The biologically active form of IL-22 is a monomer. However, non-covalent and non-intertwining dimers and high concentrations of IL-22 tetramers have also been detected (13, 14). All IL-10 family members bind to a heterodimeric receptor complex comprising two chains in the class II cytokine receptor family (CRF2) (15). IL-22 binds to IL-22 receptor alpha1 (IL-22RA1) expressed in epithelial cells, keratinocytes, and IL-10RB (16, 17) and transmits cellular signals *via* the JAK/STAT, AKT, ERK, SAPK/JNK, and MAPK signaling pathways (18). STAT3 is a major transcription factor in these cascades (19). Besides the transmembrane receptor complex, a single-chain secreted (soluble) receptor known as the IL-22 binding protein (IL-22BP; alternatively referred to as IL-22RA2) is also expressed. It is encoded by an IL-22RA1-independent gene (20). IL-22BP is secreted by various non-immune cells and tissues and has a stronger affinity for IL-22 than IL-22RA1 (21). IL-22 binding to IL-22BP prevents IL-22/IL-22RA1 interaction and competitively inhibits its signaling (20).

Multiple aspects of IL-22 physiology have been reported, including epithelial cell proliferation, tight junction formation, and mucus and AMP biosynthesis (19, 22, 23). In a murine model of IBD with dextran sulfate sodium (DSS)-induced bowel inflammation, IL-22 deficiency delayed healing and increased mortality. It downregulated the genes associated with anti-apoptosis regulation (*mcl1*, *survivin*, and *bcl2*), epithelial cell proliferation (*myc*, *pla2g5*, and *smo*), and AMP production (*S100A8*, *S100A9*, *Reg3β*, and *Reg3γ*) (24, 25). Of these, the IL-22-dependent induction of apoptosis has also been reported in recent years in the context of cell death (26). In contrast, recombinant IL-22 administration restored the production of mucins (muc-1, muc-3, muc-10, and muc-13) and goblet cell numbers that had been decreased owing to DSS-induced inflammation (19).

Teleost IL-22 has been characterized in several bony fish species and is expressed at high levels in mucosal tissues (the gills and intestines of zebrafish (*Danio rerio*) (27); gills of cod (*Gadus morhua*) and haddock (*Melanogrammus aeglefinus*) (28); gills, intestines, and tail fin of rainbow trout (*Oncorhynchus mykiss*) (29); intestines and gills of turbot (*Scophthalmus maximus*) (30); gills and intestines of golden pompano (*Trachinotus ovatus*) (31); gills and skin of yellow catfish (*Pelteobagrus fulvidraco*) (32); and gills of mandarin fish (*Siniperca chuatsi*) (33)). IL-22 was detected in rainbow trout leukocytes and epithelial cells (34). IL-22 also induces certain AMPs, such as β-defensin and hepcidin, in response to bacterial infection (29, 35). However, the roles of teleost IL-22 in the immune response and mucus homeostasis have not yet been clarified.

In this study, the *il22* and its receptors, *il22ra1* and *il22bp* cDNA sequences in Japanese medaka (*Oryzias latipes*) were characterized, and the expression in mucosal tissues using qPCR and *in situ* hybridization (ISH) was elucidated. An IL-22 knockout (KO) medaka line using CRISPR-Cas9 genome editing was newly established, and a DSS-induced inflammation model using the fish was also devised. The comprehensive transcriptomic analyses were performed by the DSS-model using IL-22-KO and wild-type (WT) medaka larvae, and their intestinal histological differences were also elucidated on epithelial repair, barrier protection, and the changes in goblet cell number and mucus layer thickness after inflammatory damage in teleosts.

## Materials and Methods

### Medaka

Healthy Japanese medaka (*Oryzias latipes*; an inbred Cab line) were maintained in transparent plastic circulating freshwater tanks at 26°C, under a 14-h light/10-h dark cycle. Both adult and larval medaka were used in this study. In the experiments on adult fish, WT fish weighing 200–300 mg at 3–4 months post-hatching (mph) were used for analyzing gene expression with respect to tissue distribution, and WT and IL-22-KO medaka weighing 100–150 mg at 2 mph were used in the DSS experiment. Larval medaka at 14 days post-hatching (dph) were used in gene expression analyses following whole-body DSS-exposed state, which is widely used in studies on the inflammatory state, similar to that in inflammatory bowel disease in mammals. All medaka were fed twice daily. The inbred Cab strain of Japanese medaka was used for *il22, il22ra1*, and *il22bp* cDNA sequence determinations and all subsequent experiments. All animal experiments were conducted in accordance with the relevant national and international guidelines, including those stated in the “Act on Welfare and Management of Animals” of the Ministry of the Environment of Japan. Ethics approval from the local Institutional Animal Care and Use Committees (IACUC) was not sought as the law does not mandate fish protection.

### Molecular Cloning of *il22*, *il22ra1*, and *il22bp* cDNAs

The Hd-rR medaka *il22, il22ra1*, and *il22bp* cDNA sequences were identified from the medaka genomic database registered in the Ensembl genome browser (https://asia.ensembl.org/index.html). The loci of *il22, il22ra1*, and *il22bp* and their adjacent synteny structures were compared among medaka, other teleosts, and mammals. To determine the *il22, il22ra1*, and *il22bp* open reading frame (ORF) sequences in Cab medaka, gene-specific primers were designed (**Supplementary Table S1**). KAPA™ HiFi-HotStart DNA (high-fidelity PCR) polymerase (Kapa Biosystems, Wilmington, MA, USA) was used in PCR amplification. The PCR products were cloned into a pTAC-2 vector (BioDynamics, Kumamoto, Japan). Plasmid DNA from ≥ three independent clones was purified using a Monarch Plasmid Miniprep kit (New England Biolabs, Ipswich, MA, USA). Sequencing was performed in a 3730xl DNA Analyzer (Applied Biosystems, Foster City, CA, USA). The aa sequences deduced for each ORF were used to predict the functional domain structures of Cab medaka IL-22, IL-22RA1, and IL-22BP using the Simple Molecular Architecture Research Tool (SMART v.7.0) (http://smart.embl-heidelberg.de/smart/set_mode.cgi?NORMAL=1). Multiple alignments of the IL-22, IL-22RA1, and IL-22BP aa sequences were performed using the multiple alignment tool ClustalW (http://www.mbio.ncsu.ebu/BioEdit/bioedit.html) in BioEdit. Signal peptide sequences were predicted using the SignalP-5.0 Server (http://www.cbs.dtu.dk/services/SignalP/). Protein structure homology modeling was performed using the SWISS-MODEL program (https://swissmodel.expasy.org). The predicted complete aa sequences of IL-22, IL-22RA1, and IL-22BP were used for constructing phylogenetic trees using the neighbor-joining method in MEGA7.0 (https://www.megasoftware.net), with 1,000 bootstrap replicates.

### RNA Extraction and cDNA Synthesis for qPCR Analysis

Total RNA was extracted from adult WT medaka brain, gills, intestines, kidneys, liver, muscles, skin, and spleen for analyzing the tissue distribution of *il22, il22ra1*, and *il22bp* expression (n=5). In WT and IL-22-KO medaka, total RNA was extracted from the whole body of larval medaka (n=7) and the mucosal tissues (from the anterior intestine, posterior intestine, gills, and skin) of adult medaka (n=5). The comparisons between WT and IL-22-KO medaka were performed not only in the naïve sate but also in the DSS-stimulated state. For RNA extraction, the RNAiso Plus kit (Takara Bio, Kusatsu, Shiga, Japan) was used according to the manufacturer’s instructions. Total RNA quality was assessed using a NanoDrop spectrophotometer (Thermo Fisher Scientific, Waltham, MA, USA). Total RNA purity was evaluated using the OD_260_:OD_280_ ratio, which was confirmed to be > 1.8 for all samples. cDNA was synthesized from 500 ng of extracted total RNA per sample using the ReverTra Ace qPCR RT Master Kit with gDNA remover (Toyobo, Osaka, Japan) according to the manufacturer’s instructions.

### Expression Analysis Using qPCR

The cDNA samples were prepared as previously described in Section 2.3. Five and seven adult and larval fish were analyzed, respectively. Seven larval fish per group were used in the DSS experiment. For qPCR, gene-specific primers were designed and used to amplify the conserved *il22, il22ra1*, and *il22bp* regions.

The medaka β-actin (*actb*) gene was used as the internal control to confirm cDNA quality and quantity. The primer sequences are listed in **Supplementary Table S1**. qPCR was conducted in triplicate in a 15 μL reaction volume comprising 7.5 μL of Brilliant III Ultra-Fast SYBR^®^ Green QPCR Master Mix (Agilent Technologies, Santa Clara, CA, USA), 1.0 μL of cDNA, 1.5 μL of each forward and reverse primer (5 pmol), and 3.5 μL of distilled water. The qPCR cycle was as follows: 95°C for 15 s; 60°C for 30 s; and 40 cycles on a CFX Connect™ (Bio-Rad Laboratories, Hercules, CA, USA). A melting curve analysis was performed on the amplified products at the end of each cycle to confirm amplification specificity. The relative expression ratios were calculated using the comparative threshold cycle (Ct or 2^-ΔΔCt^) method (36). The Ct values of the target gene and internal control were determined for each sample. The average Ct for triplicate samples was used to calculate the expression levels relative to that of *actb*. Student’s *t*-test was used when homoscedasticity between group pairs could be assumed. Welch’s *t*-test was used when homoscedasticity between group pairs could not be assumed.

### ISH

ISH was performed on adult medaka (3 mph) gills and intestines and larval medaka (14 dph) intestines to evaluate the localization of *il22* and *il22bp* mRNA. A gene-specific digoxigenin (DIG)-labeled RNA probe was synthesized with gene-specific primers (to amplify the full-length ORF; **Supplementary Table S1**) using a DIG RNA labeling kit (SP6/T7; Roche Diagnostics, Basel, Switzerland) according to the manufacturer’s instructions. Briefly, tissue samples were fixed overnight in 4% (v/v) paraformaldehyde (PFA)/0.1 M phosphate buffer (PB) at 4°C. The tissue samples were dehydrated, embedded in paraffin (Fujifilm Wako, Osaka, Japan), and cut into 8 μm-thick sections using a microtome (Leica Biosystems, Wetzlar, Germany). After dewaxing and rehydration, the sections were permeabilized with proteinase K (Fujifilm Wako) in diethyl pyrocarbonate (DEPC)-treated phosphate-buffered saline (PBS) (5 μg/mL) at 37°C for 15 min, fixed in 4% (v/v) PFA/PBS for 10 min, and treated twice with DEPC-treated PBS containing glycine (2 mg/mL) for 10 min. The sections were post-fixed with 4% (v/v) PFA/0.1 M PB for 5 min. Prehybridization was performed for 2 h using a probe diluting solution (50% (v/v) formamide, 5× SSC, 5× Denhardt’s solution (Fujifilm Wako), and 2 mg/mL RNA (Roche Diagnostics) in DEPC-treated water) after 30 min of incubation in 5× SSC/formamide. The DIG-labeled antisense and sense RNA probes were diluted using a probe-diluting solution (0.5 μg/mL), and hybridization was performed at 55°C for 16 h. DIG was detected using horseradish peroxidase-labeled anti-DIG immunoglobulin G (IgG), and color was developed using nitro-blue tetrazolium chloride and 5-bromo-4-chloro-3’-indolyphosphate p-toluidine salt (NBT/BCIP) solution (Roche Diagnostics).

### IL-22-Deficient Medaka Strain Establishment

Benchling (https://www.benchling.com/academic/) was used to design a crRNA in exon 1 of medaka *il22*. The crRNA sequence is shown in **Supplementary Figure S5A**. The sgRNA was prepared by annealing the crRNA and tractr-RNA (Thermo Fisher Scientific). Approximately 0.5 nL of a solution containing sgRNA (50 ng/μL) and Cas9 protein (400 ng/μL) (Thermo Fisher Scientific) was co-injected with a manipulator (Narishige, Tokyo, Japan) into single-cell-stage medaka embryos. Medaka large eggs facilitates microinjection during genome editing (37). One month later, the gene editing efficiency of the extracted genomic DNA was confirmed in a heteroduplex mobility assay (HMA) using a primer set (**Supplementary Figure S1**) amplifying the crRNA and other specific regions. F0 medaka with confirmed mutations were interbred with WT medaka (Cab) to produce F1 heterozygotes. The latter were then interbred with WT Cab medaka to produce F2 heterozygotes. F2 medaka males and females with the same mutation were mated to produce F3 homozygous progeny and/or mutant lines. HMA verified the mutant locus in the F3 medaka genome. Briefly, F3 medaka were anesthetized with MS-222 (Sigma-Aldrich, St. Louis, MO, USA), and their genomic DNA was extracted from the epidermal mucosa, dissolved in 20 μL of 0.2 mM EDTA (Fujifilm Wako) and 25 mM NaOH (Fujifilm Wako), and incubated at 95°C for 20 min. The samples were then neutralized with an equal volume of 40 mM Tris/HCl (pH 8.0) (FUJIFILM Wako). The genomic DNA-containing solution was used as a template, and PCR was performed using KOD FX Neo (Toyobo). The PCR conditions were as follows: 95°C for 3 min; 38 cycles of 98°C for 10 s, 66°C for 5 s, and 68°C for 5 s; and 72°C for 5 min. The PCR products were cloned into the pTAC-2 vector (BioDynamics). Polymerase DNA from ≥ three independent clones was purified using a Monarch Gibraltar Miniprep kit (New England Biolabs). Sequencing was performed in a 3730xl DNA Analyzer (Applied Biosystems).

### DSS Inflammation Model

Medaka larvae at 14 dph were used for the DSS exposure test. WT and IL-22-KO medaka larvae were obtained by natural spawning and raised until 7 dph at 26°C in freshwater supplemented with methylene blue. The larvae were then transferred to plain freshwater until the experiment commenced.

Inflammation was induced with 0.5% (w/v) DSS (40,000 MW; Sigma-Aldrich) per a previously described method (38). DSS stock solution (10% w/v) was diluted to 0.5% (w/v) in freshwater at 26°C with gentle rocking. Larval medaka (14 dph) were stimulated with 0.5% (w/v) DSS for 24 h and transferred to breeding water. Samples were collected on day 1 for histological and transcriptomic analyses and again on days 2 and 5 for histological analyses.

### Next-Generation Sequencing (RNA-Seq)

For RNA-seq analysis, WT and IL-22-KO larval medaka (14 dph) under naïve conditions and 1-day DSS stimulation were compared. Total RNA was extracted from whole larval Cab Japanese medaka using the RNAiso Plus Kit (Takara Bio) according to the manufacturer’s instructions. Total RNA from each medaka larva was extracted separately and not normalized. RNA was quantified using a NanoDrop spectrophotometer (Thermo Fisher Scientific) at OD_260_:OD_280_. A ratio of 1.8 was set as the minimum RNA purity cut-off. To synthesize the cDNA library, total RNA from ten individuals per group were equally pooled and sequenced in a DNBSEQ-G400 instrument (Mouse Genome Informatics, Bar Harbor, ME, USA) using Danaform (Yokohama, Japan).

### Sequence Read Mapping and Differential Expression and Gene Enrichment Analyses

Processed reads were deposited in the DNA Data Bank of Japan (DDBJ) Sequence Read Archive under the Accession No. DRA011594. The collected reads were mapped to the annotated medaka Hd-rR reference genome (release 85; http://www. ensembl.org/index.html) using the STAR program and analyzed using its Feature Counts function. Transcriptional expression was estimated as fragments per kilobase of exon length per million reads. Transcripts with *P* < 0.05 were considered significantly differentially expressed. Genes that were significantly differentially expressed in each comparison were subjected to gene enrichment analysis using Database for Annotation, Visualization, and Integrated Discovery (DAVID) (39). Gene ontology (GO) terms in the biological process (BP) (GOTERM_BP_FAT), cellular component (CC) (GOTERM_CC_FAT), and molecular function (MF) (GOTERM_MF_FAT) categories as well as Kyoto Encyclopedia of Genes and Genomes (KEGG) pathways were selected. Gene interactions and networks were analyzed using the STRING App database of Cytoscape (version 3.8.0) (40).

### Histological Staining

Larval medaka were anesthetized by soaking in 0.2 mg/mL MS-222 and fixed overnight with Davidson’s fixative at 4°C. The samples were dehydrated with an alcohol gradient series and Hist-clear and embedded in paraffin (Fujifilm Wako). Transverse or parasagittal sections of 5 μm thickness were cut using a microtome (Leica Biosystems) and mounted on PLATINUM PRO slides (Matsunami, Osaka, Japan). H&E and AB staining were performed to identify the phenotypic differences between WT and IL-22-KO medaka as well as to observe DSS-induced inflammation. For H&E staining, the slides were dewaxed using Clear Plus, hydrated with an ethanol/water gradient, and stained with hematoxylin (Fujifilm Wako) and 1% eosin solution for 5 min. Mucin was stained with 3% (w/v) Alcian blue (Sigma-Aldrich) in 3% (v/v) acetic acid at pH 2.5 for 30 min. Histological observations were performed using BZ-X700 (KEYENCE, Osaka, Japan). In each group, three individual larvae were used for counting goblet cells (n=3), and the area corresponding to the area observed is shown in **Figure 8B**, which was confirmed to form a part of the anterior intestine in teleosts. Individually, ten consecutively sliced sections of 5 μm (total thickness of 50 μm) were used for counting. The goblet cells in the anterior intestinal epithelia were manually counted in each section, and ImageJ version 1.53a (https://imagej.nih.gov/ij/) was used for calculating the area size. For detecting apoptotic cells, terminal deoxynucleotidyl transferase dUTP nick-end labeling (TUNEL) staining was performed using the *In Situ* Cell Death Detection Kit, TMR red (Sigma-Aldrich) according to the manufacturer’s instructions. TUNEL staining is often used to assess the progression of inflammation in mammalian DSS-based inflammation models (41, 42). Three consecutively sliced sections, each of thickness 5 μm, from the anterior intestine of larval medaka were used for counting positive signals, and in each group, three larval medaka were used individually for counting. Positive TUNEL staining signals were manually counted in each section, and ImageJ version 1.53a (https://imagej.nih.gov/ij/) was used for calculating the area.

## Results

### Characterization of the *il22*, *il22ra1*, and *il22bp* cDNA Sequences of Cab Medaka

According to data in the Ensembl database, the full-length *il22* cDNA sequence of Hd-rR Japanese medaka (Ensembl ID, ENSORLG00000026810) contains 573 base pairs (bp). It contains a 573 bp ORF encoding a predicted 190 aa protein with an estimated mass of 21.30 kDa. For Cab Japanese medaka, we cloned the 570 bp ORF of *il22* cDNA (GenBank accession No. LC528229) encoding a predicted 189 aa protein with a 35 aa signal peptide at the *N*-terminus (**Supplementary Figure S1A**). The mature IL-22 peptide contains 154 aa, and its estimated mass is 17.77 kDa.

The full-length *il22ra1* cDNA sequence of Hd-rR Japanese medaka (ENSORLG00000027190) is of 4,144 bp and contains a 1,539 bp ORF encoding a predicted 512 aa protein with an estimated mass of 56.72 kDa. For inbred Cab Japanese medaka, a 1,539 bp ORF of *il22ra1* cDNA (LC528230) was cloned, which encoded a predicted 512 aa protein with a 22 aa signal peptide at the *N*-terminus (**Supplementary Figure S1B**). The mature IL-22RA1 peptide contains 490 aa, and its estimated mass is 54.25 kDa.

The full-length *il22bp* cDNA sequence of Hd-rR Japanese medaka was of 1,717 bp and comprised a 660 bp ORF encoding a predicted 219 aa protein with an estimated mass of 24.87 kDa (ENSORLG00000019053). For the inbred Cab Japanese medaka, we cloned a 660 bp ORF of the *il22bp* cDNA (LC528231) encoding a predicted 219 aa protein with a 21 aa signal peptide at the *N*-terminus (**Supplementary Figure S1C**). The mature IL-22BP peptide contains 198 aa, and its estimated mass is 22.72 kDa.

### Putative Functional Domain and Motif Comparisons

Multiple sequence alignments revealed substantial conservation among the predicted aa sequences and functional domains of medaka IL-22, IL-22RA1, and IL-22BP and those of other organisms (**Supplementary Figure S1**). Japanese medaka Ol_IL-22 contains four cysteine residues, which are conserved in IL-22 from other fish species. Of the four cysteine residues, three are also conserved in mammals. The Ol_IL-22 sequence showed a high identity (66.8%) and similarity (87.8%) with the Chinese perch Sc_IL-22 sequence in GenBank. The six A–F α-helices present in Human Hs_IL-22 were also identified in Ol_IL-22 (**Supplementary Figure S1A**). According to PSIPRED (http://bioinf.cs.ucl.ac.uk/psipred/) and SWISS-MODEL programs, the sequence features and predicted 3D structure locations of Ol_IL-22 resembled those of its human ortholog (**Supplementary Figure S2A**). The deduced Ol_IL-22 sequence contained three cysteine residues conserved among fish and mammals and one cysteine residue conserved only among fish species.

The Ol_IL-22RA1 protein has a fibronectin type III (FNIII) domain 1 (24–112 aa), a FNIII domain 2 (121–222 aa), and a transmembrane region (227–248 aa). Ol_IL-22RA1 shared similarity (64.6%) with human Hs_IL-22RA1 and showed the highest similarity (72.2%) with Japanese pufferfish Tr_IL-22RA1, among sequences from aligned species. IL-22RA1 3D structure prediction revealed structural similarity with the proteins from medaka, yellow catfish, and humans (**Supplementary Figures S1B, S2B**).

Ol_IL-22BP contained the FNIII domain 1 (28–122 aa) and the FNIII domain 2 (128–218 aa). Two FNIII domains and four cysteine residues for disulfide bridge formation were conserved in all aligned IL-22BP proteins. Ol_IL-22BP shared similarity (66.7%) with human Hs_IL-22BP and showed the highest similarity (75.36%) with Atlantic salmon Ss_IL-22BP (**Supplementary Figure S1C**). The predicted 3D structure of Ol_IL-22BP showed similarity with those of yellow catfish and humans (**Supplementary Figure S2C**).

### Phylogenetic Analyses

**Figure 1:**
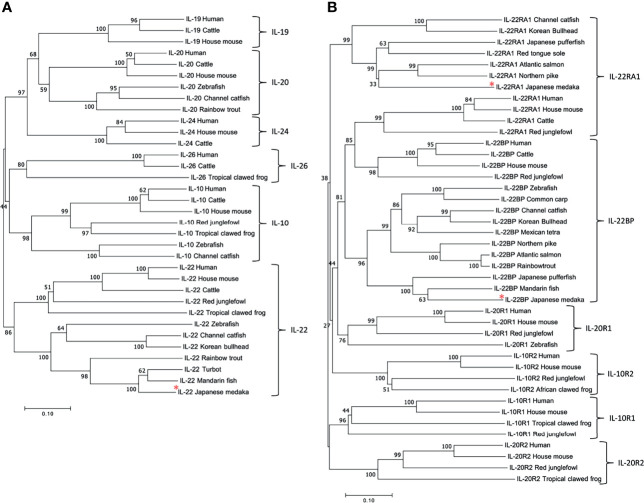
Phylogenetic tree of IL-10 cytokine family members **(A)** and cognate receptors **(B)** in different species. The tree was constructed using the neighbor-joining method. The number indicates the bootstrap confidence values obtained for each node after 1,000 replications, and the red asterisks indicate IL-22, IL-22RA1, and IL-22BP of Japanese medaka. GenBank accession numbers for all sequences are listed in **Table S2**.

We aligned the aa sequences of Japanese medaka IL-22 and members of the IL-10 cytokine family with those of other fish and vertebrates. The phylogenetic tree showed that all sequences clustered into three major clades. The first comprised smaller clades for IL-19, IL-20, and IL-24. The second comprised clades for IL-26 and IL-10. The third included a major clade subdivided into a smaller clade for IL-22 that was further divided into clades for fish, mammals, birds, and amphibians. Medaka IL-22 was localized to the fish IL-22 clade, and its nearest relatives were those of mandarin fish and turbot (**Figure 1A**).

We also aligned the aa sequences of Japanese medaka IL-22RA1, IL-22BP, and CRF2 with those of other fish and vertebrates (**Figure 1B**). In the phylogenetic tree, all sequences clustered into a clade comprising IL-10R2, IL-20R1, IL-22RA1, and IL-22BP and another comprising IL-10R1 and IL-20R2. Medaka IL-22RA1 was localized to the fish IL-22RA1 clade and was closely related to northern pike and Atlantic salmon IL-22RA1. Medaka IL-22BP was localized to the fish IL-22BP clade and closely related to mandarin fish IL-22BP.

### Synteny

We analyzed the gene order and orientation using BLASTn for the contigs harboring *il22*, *il22ra1*, and *il22bp* (**Supplementary Figure S3**). *ifng*, *mdm1*, and *cand1* were localized upstream or downstream of *il22* on chromosome 23 in Japanese medaka as well as in other fish and vertebrate species (**Supplementary Figure S3A**). *cnr2*, *pnrc2*, and *mym3* were localized upstream of *il22ra1* on chromosome 16 in Japanese medaka as well as in other fish and vertebrate species (**Supplementary Figure S3B**). *olig3* and *ifngr1* were localized upstream of *il22bp* on chromosome 15 in Japanese medaka as well as in other fish and vertebrate species, except zebrafish (**Supplementary Figure S3C**).

### Tissue Distribution of *il22*, *il22ra1*, and *il22bp* mRNA

**Figure 2:**
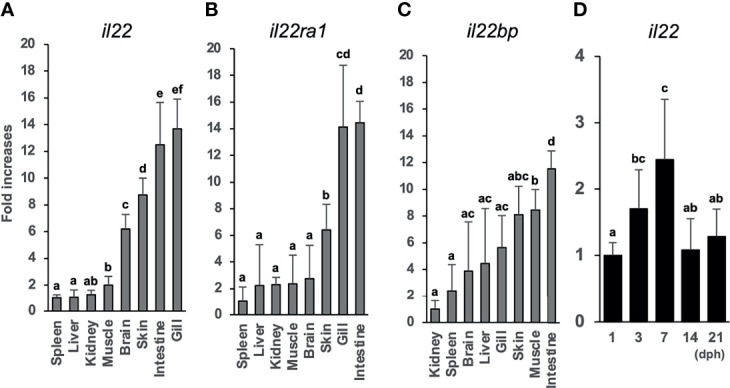
Tissue distributions of *il22* **(A)**, *il22ra1* **(B)**, and *il22bp* **(C)** mRNAs in the inbred adult Japanese medaka Cab strain. *il22* expression levels in adult Japanese medaka Cab strain **(D)**. The expression levels were determined using qPCR and normalized against the β-actin (*actb*) expression levels. **(A–C)** The expression scale reflects the relative values when the value for the tissue with lowest expression was set to 1, and the expression levels were arranged from the left in ascending order. **(D)** The expression scale shows relative values when the value for the 1 day post-hatching group was set to 1. Different letters above the bars indicate significant difference at p<0.05, according to the Tukey-Kramer multiple comparisons tests after one-way analysis of variance. Bars represent mean ± standard error (n = 5).

**Figure 3:**
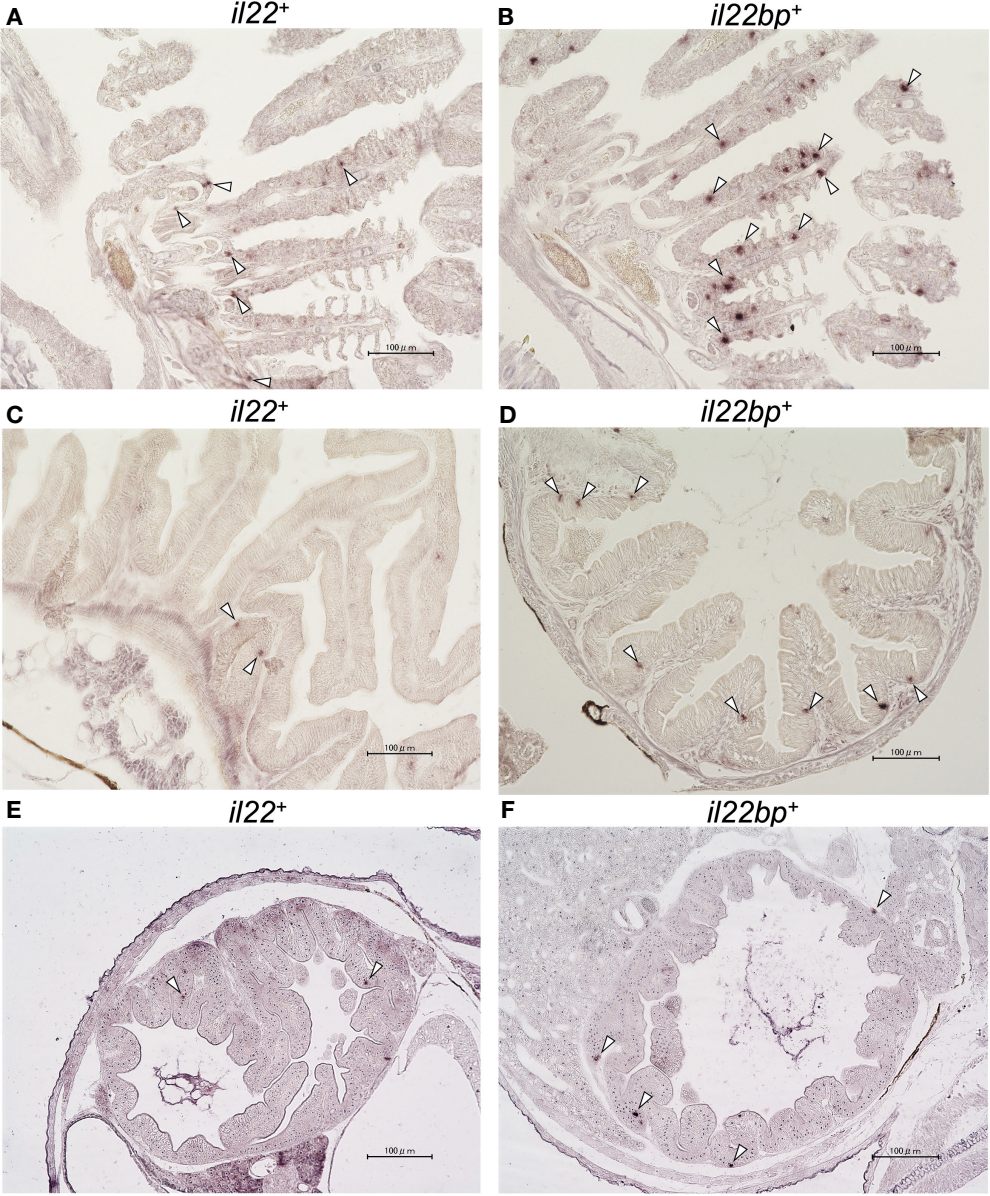
*In situ* hybridization (ISH) of medaka *il22* and *il22bp* mRNA localization. *il22* **(A)** and *il22bp* **(B)** expression in adult medaka gill; *il22* **(C)** and *il22bp* **(D)** expression in adult medaka intestine; *il22* **(E)** and *il22bp* **(F)** expression in larval medaka intestine. Scale bar: 100 μm. The white arrowheads indicate positive *il22* and *il22bp* mRNA signals in the gill and intestinal epithelia. After 4% (v/v) paraformaldehyde fixation, the gills and intestines of healthy adult medaka and the whole body of larvae were embedded in paraffin. Digoxigenin (DIG)-labeled anti-sense RNA-probes were used for detection. After hybridization, color development was performed using AP-labeled anti-DIG IgG (sheep) and NBT/BCIP solution.

We analyzed the *il22*, *il22ra1*, and *il22bp* expression levels in the brain, gills, intestines, kidneys, liver, muscles, skin, and spleen. qPCR analysis showed the expression of these genes in all sampled tissues (**Figures 2A–C**). The genes were highly expressed in the healthy medaka gills, intestines, and skin mucosae. *il22bp* was also abundant in the muscle, liver, and brain (**Figure 2C**). We also investigated temporal changes in larval (1–21 dph) *il22* expression and found that *il22* was ubiquitously expressed at all developmental stages, with significant increase in expression at 7 dph (**Figure 2D**). We performed histological staining by ISH on adult medaka (3 mph) gills and intestines and larval medaka (14 dph) intestines. We observed *il22* and *il22bp* expression in the intestinal and gill epithelia of healthy medaka (**Figures 3A–D**) and the intestinal epithelia of medaka larvae (**Figures 3E, F**), with no detection in the negative controls using sense-probe (**Supplementary Figure S4**). Even though we attempted to detect *il22ra1* expression, the signal was not observed (data not shown).

### IL-22-KO Medaka Strain Establishment

The crRNA in exon 1 of *il22* was highly mutated (**Figure S5D**). We injected a mixture of sgRNA and Cas9 protein into the embryo and confirmed a 4 bp deletion in the region containing the crRNA (**Supplementary Figures S5B, E**). In the mutant strain, the IL-22 aa sequence was terminated in the middle of the full-length sequence because of a codon frameshift (**Supplementary Figure S5C**). In contrast, the IL-22-KO (–4) larval and adult strains showed no morphological anomalies (**Supplementary Figure S6**).

### *il22*, *il22ra1*, and *il22bp* Expression in Larval WT and IL-22-KO Medaka

Compared with that in WT, in IL-22 (–4)-KO medaka, *il22* (*i.e.*, mutated *il22* transcripts) and *il22bp* were markedly downregulated (**Supplementary Figures S7A, C**). There was no difference in *il22ra1* expression between IL-22-KO and WT (**Supplementary Figure S7B**).

### Larval WT and IL-22-KO Medaka Transcriptome Analysis

**Figure 4:**
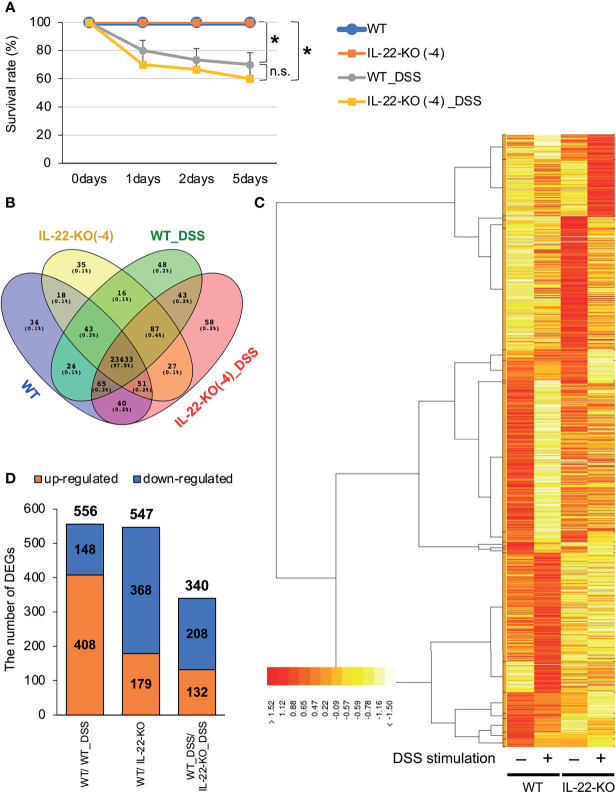
RNA-sequencing (RNA-seq) analysis of larval WT and IL-22-KO medaka samples treated with dextran sulfate sodium (DSS). **(A)** Survival analysis of larvae (14 days post-fertilization) stimulated with DSS (n ≥ 10/group; six biological replicates). Error bars indicate 95% confidence intervals (CIs), black asterisks indicate significant differences in survival rates, and “n.s.” indicates no significant change. WT *vs.* WT_DSS; WT *vs.* IL-22-KO; WT_DSS *vs.* IL-22-KO_DSS: *P* < 0.05 determined using the log-rank test. **(B–D)** Overall gene expression patterns for WT, IL-22-KO, WT_DSS, and IL-22-KO_DSS. **(B)** The Venn diagram shows the number of expressed and overlapping genes per group. **(C)** Heat map showing the differences in overall gene expression patterns among groups constructed using the TCC-GUI software. **(D)** Number of differentially expressed genes (DEGs) detected in WT/IL-22-KO, WT/WT_DSS, and WT_DSS/IL-22-KO_DSS; 556 DEGs were identified between WT and IL-22-KO, 547 between WT and WT_DSS, and 340 between WT_DSS and IL-22-KO_DSS.

WT and IL-22-KO medaka (14 dph) were treated with 0.5% (w/v) DSS for 24 h and observed for 5 days to confirm whether DSS caused reproducible inflammation. DSS stimulation drastically lowered the relative survival rates. Nevertheless, there was no significant difference between WT and IL-22-KO medaka in terms of post-DSS treatment survival (**Figure 4A**).

**Figure 5:**
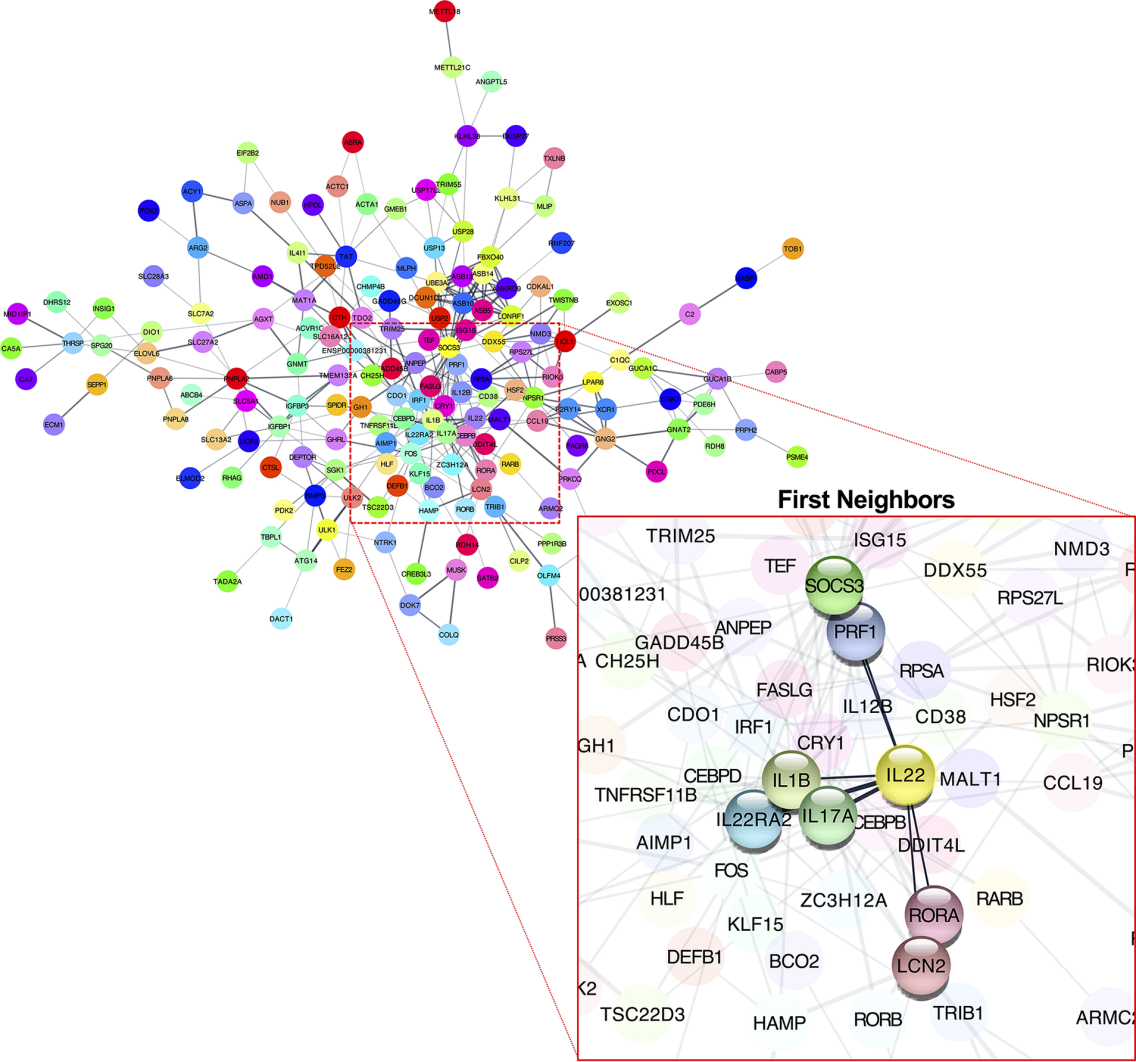
Predicted interactions among downregulated differentially expressed genes in WT and IL-22-KO medaka. Interaction network between genes defined using the STRING App database (Cytoscape). Clusters included IL-22. The gene cluster included *rora*, *il1β*, *il17a/f1*, *il22bp*, *socs3*, *lcn*, and *prf1*.

**Figure 6:**
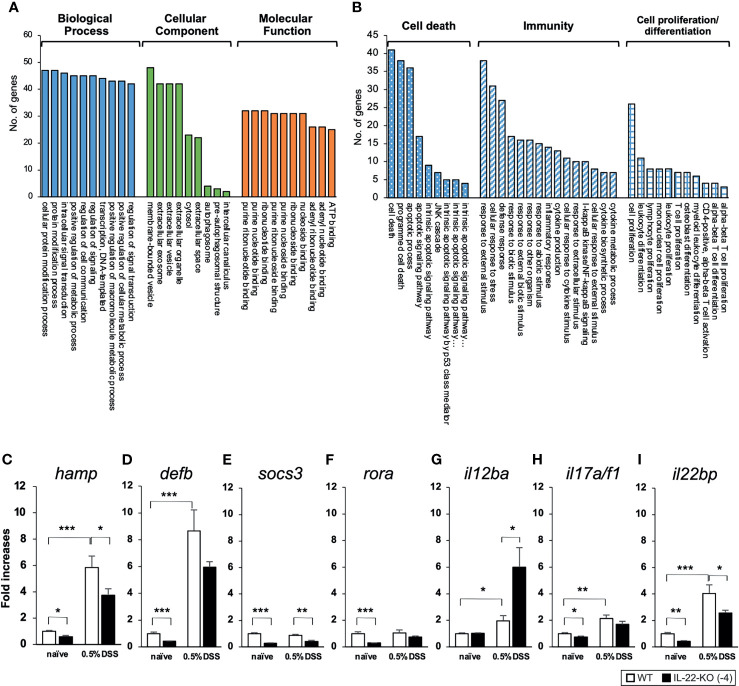
Gene ontology (GO) enrichment classifications of downregulated differentially expressed genes (DEGs) between WT and IL-22-KO medaka. **(A)** Top 10 terms with the highest number of genes in the biological process (BP), cellular component (CC), and molecular function (MF) categories. **(B)** Downregulated DEGs related to GO terms describing immunity, cell death including apoptosis, and cell proliferation/differentiation under BP. Among immune-related DEGs, the expression levels of **(C)** *hamp*, **(D)** *defb*, **(E)** *socs3*, **(F)** *rora*, **(G)** *il12ba*, **(H)** *il17a/f1*, and **(I)** *il22bp* were confirmed using qPCR. ***P < 0.001, **P < 0.01, *P < 0.05, +;*P* < 0.1 (two-tailed Student’s *t*-test). **(C-I)** The expression scale reflects the relative the values when the expression of the naïve WT group was set to 1. Data shown were obtained from a single experiment (n = 7).

We performed RNA-seq to investigate the effects of IL-22-KO on IL-22 downstream gene expression in response to DSS-induced inflammation. We obtained averages of 208,381,750 (WT), 218,140,591 (IL-22-KO), 210,655,244 (WT_DSS), and 217,765,749 (IL-22-KO_DSS) reads from the synthesized cDNA library. After annotation, 23,708 (WT), 23,710 (IL-22-KO), 23,759 (WT_DSS), and 23,804 (IL-22-KO_DSS) genes were detected in each library (**Figure 4B**). The overall gene expression differed between WT and IL-22-KO and between DSS-treated and untreated fish (**Figure 4C**). There were 179 upregulated and 368 downregulated genes in IL-22-KO compared to that in WT (**Figure 4D**). **Supplementary Table S3** lists the top 50 differentially expressed genes (DEGs) between WT and IL-22-KO. In protein-protein interaction network analyses using the STRING App in Cytoscape, 170 of 368 downregulated genes in IL-22-KO formed a series of cluster with IL-22. Of these, *rora*, *il1b*, *il17a/f1*, *il22bp*, *socs3*, *ptgdsb.1* (*lcn)*, and *prf1* were confirmed as the first interactors with IL-22 (**Figure 5**). GO analysis was performed on the significantly downregulated genes using DAVID. **Figure 6A** shows the top 10 terms under BP, CC, and MF with most genes. Under BP, terms related to various types of immunity, cell death (including apoptosis), and cell proliferation/differentiation were annotated (**Figure 6B**). Immune-related genes, including *socs3* and *rora*, and those encoding AMPs (*defb* and *hamp*) and cytokines (*il1β*, *il12ba*, *il17a/f1*, and *il22bp*) also interacted with mammalian IL-22. qPCR confirmed that the genes were downregulated in IL-22-KO medaka (**Figures 6C–I**).

### DSS-Induced Injury and Repair in WT and IL-22-KO Medaka

**Figure 7:**
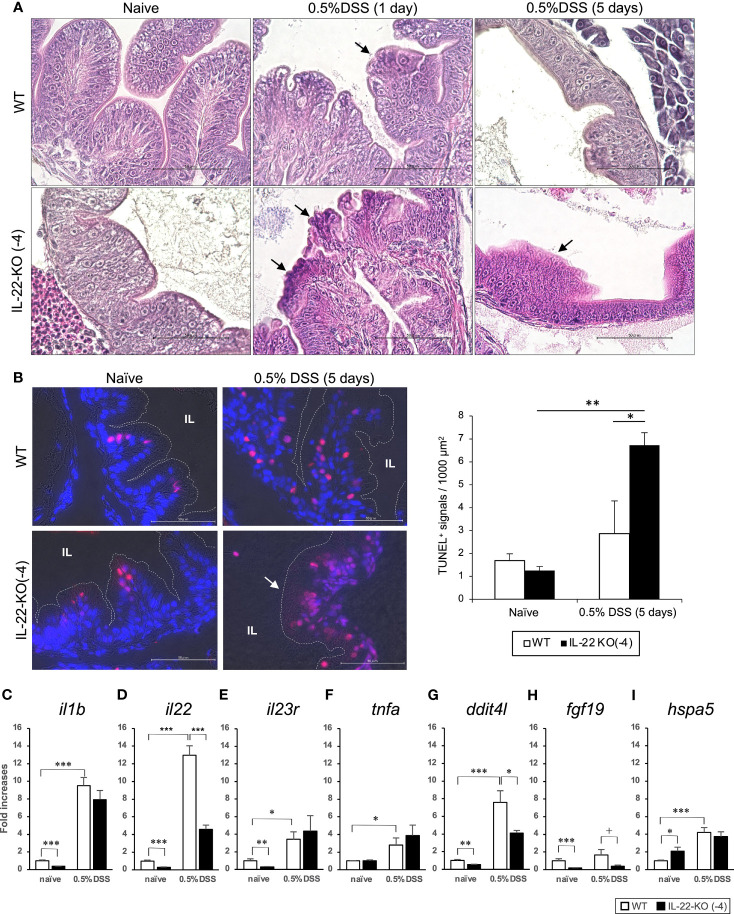
Dextran sulfate sodium (DSS)-induced inflammation in WT and IL-22-KO medaka. **(A)** Hematoxylin/eosin (H&E)-stained anterior intestinal tissue section of WT and IL-22-KO medaka larvae at 1 and 5 days after DSS stimulation. Arrows indicate the changes in tissue architecture upon DSS stimulation. **(B)** TUNEL staining in the anterior intestine corresponding to the area observed in H&E staining. The red/pink signals indicate the fragmented nuclear signals by apoptosis, and the blue signals represent nuclear staining by DAPI. The red/pink signals were counted in three consecutive sections for three individual larvae (n=3) from each group and were also counted manually under a microscope. IL: intestinal lumen. **(A, B)** Scale bar: 50 μm. **(C–I)** Inflammatory bowel disease-sensitive inflammatory cytokine and PI3K-Akt and MAPK signaling pathway genes were significantly downregulated to < 50% of that in DSS-stimulated IL-22-KO medaka, as confirmed by qPCR. **(C)** *il1b*, **(D)** *il22*, **(E)** *il23r*, **(F)** *tnfa*, **(G)** *dditl4*, **(H)** *fgf19*, and **(I)** *hspa5*. ***P < 0.001, **P < 0.01, *P < 0.05, +; *P* < 0.1 (two-tailed Student’s *t*-test). **(C–I)** The expression scale shows the relative values when the expression of the naïve WT group was set to 1. Data shown were obtained from a single experiment (n = 7).

H&E staining was performed for WT and IL-22-KO medaka at 1, 2, and 5 days after DSS stimulation to detect intestinal injury and regeneration. We observed erosion in the anterior intestines of WT and IL-22-KO medaka at 1 and 2 days after DSS stimulation (**Figures 7A** and **Supplementary Figure S8**). At 5 days after DSS exposure, the intestinal epithelium was regenerated in the anterior intestine of WT medaka (**Figures 7A** and **Supplementary Figure S8**). In contrast, the anterior intestine, particularly the damaged villi (indicated by black arrows), did not recover in IL-22-KO medaka (**Figures 7A** and **Supplementary Figure S8**). TUNEL staining was performed for assessing the degree of apoptosis caused by DSS. Under naïve conditions, the number of positive cells between WT and IL-22-KO did not differ significantly, and both groups showed signals derived from the neoplasia of the intestinal epithelium on the top of the villi. Consistent with the H&E-based observation at 5 days after DSS stimulation, TUNEL-positive cells became ubiquitous in the intestinal epithelium, and the number of TUNEL-positive cells per field in IL-22-KO medaka was significantly higher than that in WT medaka (**Figure 7B**). We then subjected the genes and IBD-sensitive proinflammatory cytokine genes—*il1b*, *il22*, *il23r*, and *tnfa—*to qPCR to confirm the expression levels. *il1b*, *il22*, *il23r*, and *tnfa* were significantly upregulated in response to DSS stimulation compared to that in untreated tissues (**Figures 7C–F**). Of the genes, *il22* was significantly downregulated in IL-22-KO medaka compared to that in WT medaka (**Figure 7D**).

For RNA-seq, we compared relative changes in the gene expression of WT and IL-22-KO medaka treated with DSS. There were 556 significant DEGs between WT and WT_DSS (*P* < 0.05). **Supplementary Table S4** lists the top 50 DEGs between WT and WT_DSS. Of these, 408 were upregulated and 148 were downregulated (**Figure 4D**). However, a comparison of WT_DSS and KO_DSS showed 340 significant DEGs (*P* < 0.05). **Supplementary Table S5** lists the top 50 DEGs between WT_DSS and KO_DSS. In all, 132 genes were upregulated, and 208 genes were downregulated (**Figure 4D**). GO analysis was performed on the DEGs between WT and WT_DSS. The 148 downregulated genes annotated with terms such as lipid metabolism, defense response, and extracellular matrix organization under BP. The 408 upregulated genes annotated with terms such as cell death, acute inflammatory response, cell proliferation, angiogenesis, cell growth, cell migration, cell-junction, and Wnt signaling pathway (**Table 1**). KEGG pathway enrichment analysis showed that the PI3K-Akt and MAPK signaling pathways were enhanced (**Supplementary Figures S9** and **S10**). GO analysis was performed on the DEGs obtained by comparing WT_DSS and IL-22-KO_DSS. The 208 downregulated genes annotated with terms such as response to chemical stimulus, complement activation, cell growth, and angiogenesis (**Table 1**). KEGG pathway enrichment analysis showed that the PI3K-Akt and MAPK signaling pathways were inhibited (**Supplementary Figures S9** and **S10**). In contrast, the 132 upregulated genes annotated with terms related to cell death and cytokine secretion (**Table 1**).

**Table 1:** List of the differentially expressed genes (DEGs) in the biological process (BP) category by Gene Ontology (GO) analyses.

Terms in the BP category	Gene names*	No. of genes**
**Downregulated: WT/WT_DSS (29 genes out of a total of 148 genes)**		
lipid metabolic process (GO: 0006629)	*ch25h, neu3, plb1, ptgr1, rdh8, enpp7, nr5a2, hsd17b7, apod, rora, apod*	17
regulation of defense response (GO: 0031347)	*il12b, ca7, apod, samhd1, rora, apod*	7
extracellular matrix organization (GO: 0030198)	*ctss, mmp13, gfap, cd36*	5
**Up-regulated: WT/WT_DSS (171 genes out of a total of 408 genes)**		
cell proliferation (GO: 0008283)	*kmt2d, nrp2, nr4a3, cd38, sphk1, birc6, ahr, ccl19, il1b, tgm1, odc1, hipk2, klf13, nacc2, itgb3, ago3, carmil2, shc4, dicer1, speg, eif4g1*	35
cell growth (GO: 0016049)	*kmt2d, nrp2, cd38, sphk1, cacna1a, unc13a, itgb3, eif4g1*	11
positive regulation of cell migration (GO: 0030335)	*nrp2, sphk1, ccl19, onecut2, itgb3, hspa5, carmil2, b3gnt3*	10
lymphocyte proliferation (GO: 0046651)	*cd38, ahr, ccl19, il1b, carmil2*	8
mononuclear cell proliferation (GO: 0032943)	*cd38, ahr, ccl19, il1b, carmil2*	8
leukocyte proliferation (GO: 0070661)	*cd38, ahr, ccl19, il1b, carmil2*	8
T cell proliferation (GO: 0042098)	*ccl19, il1b, carmil2*	6
Wnt signaling pathway, calcium modulating pathway	*tnrc6c, ago3*	4
cell-substrate adhesion (GO: 0031589)	*mslnl, lyve1, ecm2, onecut2, tecta, itgb3, srcin1, tecta*	9
cell junction organization (GO: 0034330)	*gjd3, itgb3, nfasc*	8
regulation of cell death (GO: 0010941)	*nr4a3, cd38, sphk1, ccl19, dnmt3a, il1b, cacna1a, thra, hipk2, prkaa2, nacc2, gimap8, hspa5, mkl1, grin1*	28
vasculature development (GO: 0001944)	*nrp2, rnf213, sphk1, ahr, il1b, hipk2, epas, c6, itgb3, anpep*	17
angiogenesis (GO: 0001525)	*nrp2, rnf213, sphk1, il1b, hipk2, epas, c6, itgb3, anpep*	12
regulation of acute inflammatory response (GO: 0002673)	*c4b, il1b, c6*	4
positive regulation of NF-kappaB import into nucleus (GO: 1901224)	*sphk1, ccl19, il1b*	3
**Down-regulated: WT_DSS/IL-22-KO_DSS (87 genes out of a total of 208 genes)**		
cellular response to chemical stimulus (GO: 0070887)	*fgf19, hyal2, nr4a3, slc25a33, tmem100, nfe2l1, ddit4l, serpine1*	24
response to external stimulus (GO: 0009605)	*hyal2, tnip1, nr4a3, klf10, c6, ddit4l, serpine1*	20
regulation of response to stress (GO: 0080134)	*fgf19, hyal2, tnip1, nr4a3, c6, serpine1, usp13*	14
regulation of cell growth (GO: 0001558)	*tmem100, c6, serpine1*	6
regulation of vasculature development (GO: 1901342)	*serpine1*	5
blood coagulation (GO: 0007596)	*c6, serpine1*	5
positive regulation of angiogenesis (GO: 0045766)	*c6, serpine1*	4
cytokine secretion (GO: 0001816)	*hyal2*	4
complement activation (GO: 0006956)	*c1r, c6*	3
chemokine secretion (GO: 0001816)	*hyal2, slc25a33*	2
**Up-regulated: WT_DSS/IL-22-KO_DSS (23 genes out of a total of 132 genes)**		
cell death (GO: 0008219)	*mal, bdkrb2, cd38, bok, ccl19, spdef, klk8*	12
regulation of apoptotic process (GO: 0042981)	*mal, bdkrb2, cd38, bok, ccl19*	8
cytokine secretion (GO: 0001816)	*crp*	3

*This refers to the downregulated (< 0.5) and upregulated genes (> 2.0)r listed in this table.

**These figures indicate the hit numbers in GO term for the BP category.

Based on the RNA-seq results, we selected the PI3K-Akt and MAPK signaling pathway genes *ddit4l*, *fgf19*, and *hspa5*, which were downregulated in DSS-stimulated IL-22-KO medaka to < 50% of the level in DSS-stimulated WT medaka. Gene expression was also quantified using qPCR analysis (**Figures 7G–I**). The PI3K-Akt signaling pathway gene *ddit4l* was significantly upregulated in WT_DSS compared with that in WT, and significantly downregulated in IL-22-KO_DSS compared with that in WT_DSS (**Figure 7G**). *fgf19* was significantly downregulated in IL-22-KO medaka compared with that in WT medaka (**Figure 7H**). In contrast, qPCR analysis revealed that *hspa5* was significantly upregulated in WT_DSS compared with that in WT, but there was no significant difference in *hspa5* expression between IL-22-KO_DSS and WT_DSS (**Figure 7I**). In an additional experiment, DSS stimulation was also performed in adult medaka using immersion strategy, similar to that in larval analysis, and the genes related to inflammatory response and mucus production were selected based on the results of the larval experiment and quantified using qPCR. With respect to the qPCR analysis of *il22* and inflammation-related genes, the mucosal tissues, including those from the intestines (anterior and posterior), gills, and skin, were selected as target tissues to assess the effects of DSS. *il22* transcripts were significantly downregulated in the anterior and posterior intestines compared to that in WT in both naïve and DSS-stimulated states. The number of *il22* transcripts in the IL-22-KO medaka gills only decreased upon DSS stimulation, and no significant change was observed in the transcripts expressed in the skin. Of the investigated genes, *ddit4l* was downregulated in the IL-22-KO medaka anterior intestine in both naïve and DSS-exposed states, and this result corresponded to that of the larval experiment (**Supplementary Figure S11**).

### Complement Production in DSS-Stimulated WT and IL-22-KO Medaka

RNA-seq data analysis revealed that multiple complement genes in WT were upregulated upon DSS stimulation, and comparison of the FPKM values of WT_DSS and IL-22-KO_DSS also revealed the significantly lower expression of several complement and related genes in IL-22-KO medaka. DSS-treated WT medaka showed the significant upregulation of *c1qc*, *c1ql2*, *c4b*, and *c6* genes encoding complement factors. In IL-22-KO_DSS, however, *c1ql2*, *c1r*, *c4b*, *c5*, *c6*, and *c7* were significantly downregulated compared with that in WT_DSS. KEGG enrichment analysis showed the relative downregulation of the complement cascade in IL-22-KO_DSS (**Supplementary Figures S12A, B**). Of the abovementioned genes, the expression of *c1qc* and *c6* were also confirmed by qPCR. The expression of *c1qc* increased significantly in WT upon DSS stimulation, as observed *via* RNA-seq (**Supplementary Figure S12C**). Additionally, *c6* expression in IL-22-KO was significantly lower than that in WT in naïve states (**Supplementary Figures S12D**).

### Mucus Production in DSS-Stimulated WT and IL-22-KO Medaka

**Figure 8:**
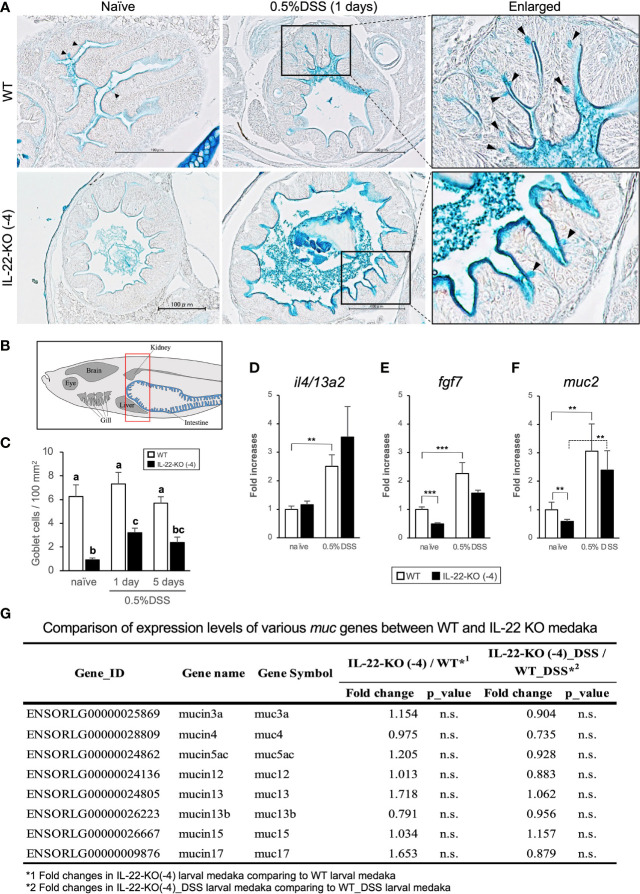
Mucin production in response to dextran sulfate sodium (DSS) stimulation in WT and IL-22-KO medaka. **(A)** Alcian blue-stained anterior intestinal tissue section of WT and IL-22-KO medaka stimulated with DSS. Black arrowheads indicate Alcian blue-positive cells (goblet cells); Scale bar: 100 μm. **(B)** The area used for counting the number of goblet cell corresponds to the area surrounded by the red box in lateral view. Counting was performed in the anterior intestine section, and the counts in each group were performed using three individual larvae (n=3). **(C)** The average number of goblet cell per 100 mm^2^ of the anterior intestine in WT and IL-22-KO medaka stimulated with DSS. Ten consecutively sliced sections 5 μm in thickness (total thickness: 50 μm) were used for counting. In these sections, all regions of the intestinal epithelia were considered for counting, and the area size was calculated using ImageJ 1.53a (https://imagej.nih.gov/ij/). Different letters above the bars indicate significant difference at *p <*0.05, according to the Tukey-Kramer multiple comparison tests after one-way ANOVA. Th expression levels of **(D)** *il4/13a2*, **(E)** *fgf7*, and **(F)** *muc2* were confirmed by qPCR. ***P < 0.001, **P < 0.01, *P < 0.05, +; *P* < 0.1 (two-tailed Student’s *t*-test). **(D–F)** The expression scale shows the relative values when the expression in the naïve WT group was set to 1. Data shown were obtained from a single experiment (n = 7). **(G)** Comparison of the expression levels of various mucin genes between WT and IL-22-KO medaka by RNA-seq analysis.

We performed AB staining to detect relative changes in the mucus layer and acidic mucus-producing cells (goblet cells) in response to DSS stimulation. WT and IL-22-KO medaka presented with expanded anterior intestine mucus layers at 1 day after DSS stimulation (**Figure 8A**). However, there was no significant increase in the number of AB-positive cells in WT medaka (**Figure 8C**). Meanwhile, IL-22-KO medaka had significantly fewer AB-positive cells than WT medaka, and the numbers increased significantly after DSS stimulation (**Figure 8C**). In mammalian intestines, FGF-7 (also known as keratinocyte growth factor; KGF) is widely known to contribute to mucus production *via* goblet cell proliferation (43). Additionally, type 2 cytokine (IL-4, IL-9, and IL-13) responses, particularly those associated with IL-4 and IL-13, are known to contribute to goblet cell development (44). In teleosts, *il4/13a2* is a counterpart of two paralogous genes, mammalian *IL4* and *IL13* (45). In larval medaka subjected to DSS stimulation, qPCR analysis revealed the upregulation of *il4/13a2* in WT, but no significant difference was observed in the expression levels between WT and IL-22-KO medaka (**Figure 8D**). *fgf7* showed significantly lower expression in larval IL-22-KO medaka than in WT medaka in naïve states (**Figure 8E**). Mucin 2 (*muc2*) is a type of secretory mucin with extremely high expression in the teleost gastrointestinal tract (46). Although the medaka *muc2* sequence is submitted as an uncharacterized protein (ENSORLG00000006006) in Ensembl Genome Browser, paralogous *muc2* sequences of multiple teleost species, including medaka (Accession. No. XM_023955731), are characterized and registered in the NCBI database (https://www.ncbi.nlm.nih.gov). qPCR analysis showed that *muc2* expression in larval IL-22-KO medaka was significantly lower than that in WT in the naïve state (**Figure 8F**). However, other types of mucins annotated based on the Ensembl reference showed no significant changes in RNA-seq results (**Figure 8G**). Changes in *fgf7* and *muc2* expression in the anterior and posterior intestines of adult medaka upon DSS stimulation were also quantified using qPCR. The expression of *fgf7* and *muc2* in IL-22-KO intestines was significantly downregulated in the naïve state. After DSS stimulation, the expression of *muc2* (in both intestines) and *fgf7* (only in the posterior intestine) was significantly lower in IL-22-KO than in WT medaka (**Supplementary Figure S13**).

## Discussion

IL-22 has been characterized in several teleost species. However, its functions in teleost immunity and mucus homeostasis have not been clarified. In this study, we characterized *il22* and its receptors *il22ra1* and *il22bp* in Japanese medaka. For the first time, we established mutant medaka *il22* using the CRISPR-Cas9 genome editing system. We also developed a DSS-induced inflammation model in medaka and elucidated the roles of teleost IL-22 by comprehensive transcriptomic analyses.

The cloned medaka IL-22 comprised six α-helices, which is a typical structure of IL-10 cytokine family members (13). The crystalline structures of human and zebrafish IL-22 showed that both IL-22 proteins have two disulfide bridges. However, two positions of the bridges did not match between the human (Cys40-Cys132 and Cys89-Cys178) (13) and zebrafish (Cys117-Cys163 and Cys118-210) proteins (47), which are conserved in mammals and teleosts, respectively. Medaka IL-22 showed a relatively high percentage of sequence similarity (77.3%) with human IL-22, and the domain structure was predicted to be similar to that of human IL-22. As with mammalian IL-22RA1 and IL-22BP, medaka and other teleost homologs of these receptor genes possess two FNIII repeats, which are commonly conserved in class II cytokine receptors (48). In medaka IL-22RA1, four cysteines form two disulfide bridges, of which one bridge is common to teleosts and mammals, and another bridge is specific to teleost IL-22RA1 (32), and these residues are also conserved. No previous study on the affinity between teleost IL-22 and IL-22RA1 has been reported to date. In medaka IL-22BP, the conservation of two disulfide bridges common to teleosts and mammals was also predicted. Therefore, characteristic cysteine residues that form disulfide bridges and are conserved in teleost genes are also conserved in the two IL-22 receptors of medaka. Additionally, similar 3D structures of IL-22, IL-22RA1, and IL-22BP, with six α-helices in IL-22 and FNIII domains in IL-22RA1 and IL-22BP, were predicted. Furthermore, the results of synteny and phylogenetic analyses strongly suggested that the three IL-22-related genes identified in Japanese medaka are orthologous to the mammalian *IL22* gene.

In qPCR analysis, medaka *il22, il22ra1*, and *il22bp* were ubiquitously expressed in all tissues, including the brain, gills, intestines, kidney, liver, muscle, skin, and spleen. Of these, mucosal tissues, such as those in the gills, intestines, and skin, showed high expression of *il22, il22ra1*, and *il22bp*. The tissue distribution of gene expression was consistent with the results previously reported in other teleost species (27–34). Meanwhile, in mammals, mucosal lymphoid tissues did not show the highest levels of *IL22* expression. For example, the highest expression of mice *IL22* was detected in the cerebellum, along with a relatively high expression in the colon (49). Meanwhile, the same transcriptome data showed the high expression of *IL22RA1* and *IL22BP* in the small and large intestines of mice (49). In our histological analysis using ISH, the signals of *il22* and *il22bp* were detected in the epithelium of the intestines and gills. The expression patterns and tissue distributions of *il22* and *il22bp* suggested the putative functions of the gene products in mucosal immunity. Meanwhile, even though we attempted to detect *il22ra1* expression, the positive signal was not observed (data not shown). *il22ra1* expression was not detected *via* RNA-seq of medaka larvae, although it was detected in qPCR analysis. In teleosts, reports showing the tissue localization of fish *il22ra1* have not been published. It is known that in mammals, *IL22RA1* is broadly expressed in the epithelial cells of mucosal tissues, with no expression in specific cells, such as hematopoietic cells (50), and the ubiquitous localization of the protein in the intestinal epithelium can be confirmed by immunohistochemistry (51). Taken together, the low expression level and expression in a wide range of tissues may have prevented the detection of medaka *il22ra1 via* ISH.

Transcriptome analyses were performed for comparing the gene expression between WT and IL-22-KO medaka using RNA-seq, following which several genes were extracted based on the RNA-seq results and their expression was quantified using qPCR. In the protein-protein interaction network analysis performed using the STRING App of Cytoscape, the significantly downregulated genes in IL-22-KO medaka under naïve states formed a cluster with *IL22*, and among them, *rora*, *il1b*, *il22bp*, *il17a/f1*, *socs3*, *ptgdsb.1* (*lcn)*, and *prf1* were the first neighbor genes of IL-22. Additionally, under comparison in naïve states, GO analysis confirmed the downregulation of the genes particularly classified under GO terms related to immune response, cell death, and cell proliferation in IL-22-KO medaka. The cytokine (*il1b*, *il12ba*, *il17a/f1*, and *il22bp*), AMP (*defb* and *hamp*), and apoptosis-related genes (*bcl2115*, *nupr1*, and *chac1*) corresponded to these GO terms. Mammalian IL-22 is generally known to induce various AMPs, including S100A7, S100A8, S100A9, β-defensin 2, RegIIIc, and RegIIIb, in mammals (25, 52–55). In previous studies on IL-22RA1 KO mice, the induction of *Bcl2115*, *Nupr1*, and *Chac1* transcripts was suggested to be associated with the IL-22/IL-22RA1 axis *via* STAT3 activation (52). Furthermore, a recombinant protein-based functional study on teleost IL-22 revealed that recombinant IL-22 can induce *defb* and *hamp* expression in rainbow trout (29), *il1b* expression in grass carp (56), and *il22bp* and *hamp* expression in mandarin fish (33). Thus, the phenotypic characteristics of IL-22-KO medaka showed several similarities with those reported in previous studies on the IL-22 induction abilities in mammals and teleosts.

In this study, we treated medaka with DSS to induce IBD-like injury. The DSS-induced inflammation model presented with idiopathic erosions and ulcers, resembling ulcerative colitis symptoms. H&E staining revealed intestinal epithelial erosion in response to DSS treatment. Upon DSS stimulation by immersion, medaka larvae showed intestinal symptoms and expression changes of multiple genes similar to those previously suggested to be associated with human IBD or IBD experimental models. Both RNA-seq and qPCR analyses in whole larval medaka showed significant upregulation of the inflammatory cytokine genes *il1b*, *tnfa*, and *il22*, and the upregulation of these genes was also quantified in the intestines of adult medaka using qPCR. The expression of IL-22 and these inflammatory cytokines also increased during the development of human IBD. *Il23r*-deficient mice lacks *Il22* expression, and IL-23R-mediated IL-22 production is considerably important for improving colitis (57, 58). Furthermore, GO analyses revealed that multiple genes related to specific GO terms, including cell proliferation, regulation of cell death, angiogenesis, and Wnt signaling pathway, among others, are upregulated upon DSS stimulation. Of the genes categorized under these GO terms, the expression of genes such as *cd38* and *nr4a* was reported to be elevated in mammalian intestines upon DSS treatment (59, 60). Additionally, DSS-induced injury promotes Wnt signaling for epithelial renewal and regeneration (61). Generally, in DSS experiments on mice, stimulation is performed by supplying DSS in drinking water (62). Meanwhile, in the immersion technique used by us, inflammatory responses, such as those reported in previous studies on mice, were observed with respect to transcripts response and intestinal histology.

After DSS immersion, IL-22-KO medaka showed different symptoms and related phenotypes compared to WT medaka. In the histological study, the intestinal tissues of WT medaka recovered 5 days after DSS exposure, whereas the intestinal tissue of IL-22-KO medaka did not heal within this period and delayed wound healing in IL-22-KO medaka was observed by H&E staining. The number of positive signals in TUNEL staining at 5 days in IL-22-KO was significantly higher than that in WT. In the mammalian intestinal tract, the IL-22/STAT3 axis is known to induce various apoptosis suppressor genes (23–25). However, contradictory effects of IL-22 as an apoptosis accelerator have also been reported in recent years. A recent report showed that colonic IL-22RA1-KO impairs apoptosis and gene inductions related to DNA repair in response to DSS and azoxymethane treatment and promotes subsequent tumor development (26). However, most details of IL-22-induced apoptosis in response to DNA damage in the intestinal tract and its anti-cancer effects are yet to be clarified. In the present study, we suggested that the consistent results from H&E staining-based histological observation and TUNEL staining indicates the attenuation of the wound healing ability in IL-22-KO medaka. Multiple previous studies on IL-22-KO mice have shown that IL-22 acts *via* STAT3 activation during the protection and wound healing of the intestinal epithelium upon DSS treatment (24, 25) Transcriptomic changes that can cause histological differences between WT and IL-22-KO medaka were confirmed in the RNA-seq analysis. KEGG analysis revealed the downregulation of the PI3K-Akt and MAPK signaling pathways in IL-22-KO medaka. In the DSS-induced colitis mice model, PI3K-Akt signaling also contributed to wound healing in the intestinal epithelium (63, 64).

We examined the alteration of mucus production in DSS-treated fish and the relationship between IL-22 cascades. Our results showed the increase in mucus production upon DSS treatment and the significant decrease in goblet cell number in IL-22-KO medaka (compared to that in WT medaka) in both of naïve and DSS-stimulated states. In our analysis, the downregulation of *fgf7* and *muc2* in IL-22-KO medaka under naïve states was confirmed in both of larval whole body and adult medaka intestines. In a study on IL-22-KO mice intestines, the increase in goblet cell number and *muc2* expression was suppressed upon intestinal helminth infection (65). Additionally, patients with ulcerative colitis showed reduced goblet cell counts and mucus thickness, and these symptoms were also observed in case of DSS-induced inflammation in mice (66). *muc2* deficiency directly results in the development of colitis in mice (67). Meanwhile, zebrafish larvae immersed DSS-water in our experiments showed a drastic increase in the apparent mucus layer with no increase in goblet cell number and *muc5* expression (40). In our transcriptome analysis, the expression of *muc*, except *muc2*, showed no significant changes. The discrepancy between the apparent mucus production and the *muc* expression levels may be attributed to the quantitative change in glycosylation at the different O-type glycosylation sites present in *muc* genes. In fact, reportedly, changes in the degree of glycosylation in the *muc* gene observed in patients with IBD promotes IBD pathogenesis (68, 69).

This study showed the strong interactions between IL-22 and complement components in response to DSS treatment. At present, most details of the IL-22-dependent activation of the complement pathway in patients with IBD or in DSS-induced inflammation models remain unknown. However, IL-22-KO mice showed lower intestinal expression of C3 than WT mice, with increasing susceptibility, when infected with *Clostridium difficile* (70). Other studies on mice have shown that C1q expression is elevated in the recovery phase after DSS exposure, and C1q-mediated Wnt signaling activation has recently been suggested to be important for tissue repair and mucosal regeneration (71). In this study, we showed that the complement genes *c1qc*, *c1ql2*, *c4b*, and *c6* were significantly upregulated in WT medaka in response to DSS stimulation. Interestingly, the complement genes, including *c1ql2*, *c1r*, *c4b*, *c5*, *c6*, and *c7*, were significantly downregulated in IL-22-KO_DSS compared to that in WT_DSS. The following two areas will be addressed with a high priority in our future studies: 1) the potential direct involvement of IL-22-mediated signals in the induction of complement factors, 2) the relationship between the increased expression of complement genes and the pathophysiology of DSS-induced enteritis.

In conclusion, we established IL-22-KO medaka and compared the phenotypes between WT and KO medaka after characterizing three medaka IL-22-related genes: *il22*, *il22bp* and *il22ra1*. The phenotypic comparisons were performed based on transcriptomic and histological analyses, and the characteristics in the naïve and DSS-stimulated states were compared. IL-22-KO medaka showed the downregulation of several genes that were previously associated with IL-22-dependent induction in mammals and teleosts. Additionally, IL-22-KO medaka showed delayed wound healing after DSS stimulation and reduction of goblet cell numbers. Along with the histological characteristics of IL-22-KO, transcriptome analysis indicated expression changes in specific genes, which may have been a causal factor in the deterioration of homeostasis in the intestinal tract. Our findings showed that the DSS experimental model developed using medaka larvae may be a viable option for basic research on IBD and also suggested the involvement of IL-22-mediated signals in the pathophysiology of enteritis in medaka.

## Data Availability Statement

The datasets presented in this study can be found in online repositories. The names of the repository/repositories and accession number(s) can be found in the article/**Supplementary Material**.

## Ethics Statement

Ethical review and approval was not required for the animal study because the ethical committee does not require fish treatment.

## Author Contributions

Conceptualization: YT, YO, HM, TK, MS, and J-iH. Methodology: YT, YO, HM, TK, NH, MW, and J-iH. Visualization: YT, YO, and J-iH. Investigation and Resources: YT and YO. Data Curation, Validation, and Formal Analysis: YT, TK, NH, MW, and J-iH. Project Administration: YT, MS, and J-iH. Supervision: MS and J-iH. Writing-Original Draft: YT, YO, and J-iH. Writing-Review & Editing: TK, MS, and J-iH. Funding Acquisition: YO, MS, and J-iH. All authors contributed to the article and approved the submitted version.

## Funding

This work was supported by a Grant-in-Aid for Scientific Research (A) and (B) from the Japan Society for the Promotion of Science (JSPS), Japan [Nos. 17H01486 and 17H03863] and a Grant-in-Aid for JSPS Research Fellows from JSPS, Japan [No. 19J14996].

## Conflict of Interest

The authors declare that the research was conducted in the absence of any commercial or financial relationships that could be construed as a potential conflict of interest.

## Publisher’s Note

All claims expressed in this article are solely those of the authors and do not necessarily represent those of their affiliated organizations, or those of the publisher, the editors and the reviewers. Any product that may be evaluated in this article, or claim that may be made by its manufacturer, is not guaranteed or endorsed by the publisher.
