# Supplementary material for: Interleukin-22 Deficiency Contributes to Dextran Sulfate Sodium-Induced Inflammation in Japanese Medaka, Oryzias latipes
Source: Front Immunol. 2021 Oct 25;12:688036. doi: 10.3389/fimmu.2021.688036 (PMC8573258; doi:10.3389/fimmu.2021.688036)
Supplement: Supplementary file 1 [file DataSheet_1.pdf]

Supplementary materials

Article information

Interleukin-22 deficiency contributes to the dextran sulfate sodium (DSS)-induced inflammation in Japanese medaka, *Oryzias latipes*

Yoshie Takahashi[1,†], Yo Okamura[2,†], Nanaki Harada[1], Mika Watanabe[3], Hiroshi Miyanishi[4], Tomoya Kono[3], Masahiro Sakai[3], Jun-ichi Hikima[3*]

[1] International Course of Agriculture, Graduate School of Agriculture, University of Miyazaki, 1-1 Gakuenkibanadai-nishi, Miyazaki 889-2192, Japan
[2] Interdisciplinary Graduate School of Agriculture and Engineering, University of Miyazaki, 1-1 Gakuenkibanadai-nishi, Miyazaki 889-2192, Japan
[3] Department of Biochemistry and Applied Biosciences, Faculty of Agriculture, University of Miyazaki, 1-1 Gakuenkibanadai-nishi, Miyazaki 889-2192, Japan
[4] Department of Marine Biology and Environmental Science, Faculty of Agriculture, University of Miyazaki, 1-1 Gakuenkibanadai-nishi, Miyazaki 889-2192, Japan

† **These authors contributed equally to this work.**
* **Correspondence to:** Jun-ichi Hikima, Ph.D. (e-mail: jhikima@cc.miyazaki-u.ac.jp)

Contents:

Fig. S1. Multiple alignments of the deduced protein sequences of IL-22 (**A**), IL-22RA1 (**B**), and IL-22BP (**C**) in Japanese medaka and other vertebrates.

Fig. S2. 3D structures of IL-22 (**A**), IL-22RA1 (**B**), and IL-22BP (**C**) in human, yellow catfish, and medaka predicted using SWISS-MODEL.

Fig. S3. Schematic representation of synteny for *il22* (**A**), *il22ra1* (**B**), and *il22bp* (**C**) in various species.

Fig. S4. Negative control (sense probe) of *in situ* hybridization (ISH) in medaka *il22* and *il22bp* mRNA localization.

Fig. S5. crRNA regions, mutated sequences, and mutation efficiency of medaka *il22*.

Fig. S6. Morphological comparison of WT and IL-22-KO medaka (-4).

Fig. S7. Gene expression analyses of *il22* (**A**), *il22ra1* (**B**), and *il22bp* (**C**) in WT and KO medaka.

Fig. S8. Histological analysis of dextran sulfate sodium (DSS)-induced inflammation in WT and IL-22-KO medaka larvae.

Fig. S9. The PI3K-Akt signaling pathway maps of differentially expression gene (DEG) hits determined using data from the Kyoto Encyclopedia of Genes and Genomes (KEGG) database.

Fig. S10. MAPK signaling pathway maps of differentially expressed gene (DEG) hits determined using data from the Kyoto Encyclopedia of Genes and Genomes (KEGG) database.

Fig. S11. Real-time PCR (qPCR) analysis of inflammatory cytokines and multiple genes related to the PI3K-Akt and MAPK signaling pathways.

Fig. S12. Complement cascade maps of differentially expressed gene (DEG) hits determined using data from the Kyoto Encyclopedia of Genes and Genomes (KEGG) database.

Fig. S13. Real-time PCR (qPCR) analysis of *fgf7* and *muc2* in the anterior and posterior intestines of adult medaka.

* **Tables S1-S5** are separately shown in the Excel files as “Supplementary tables”.

Fig. S1. A

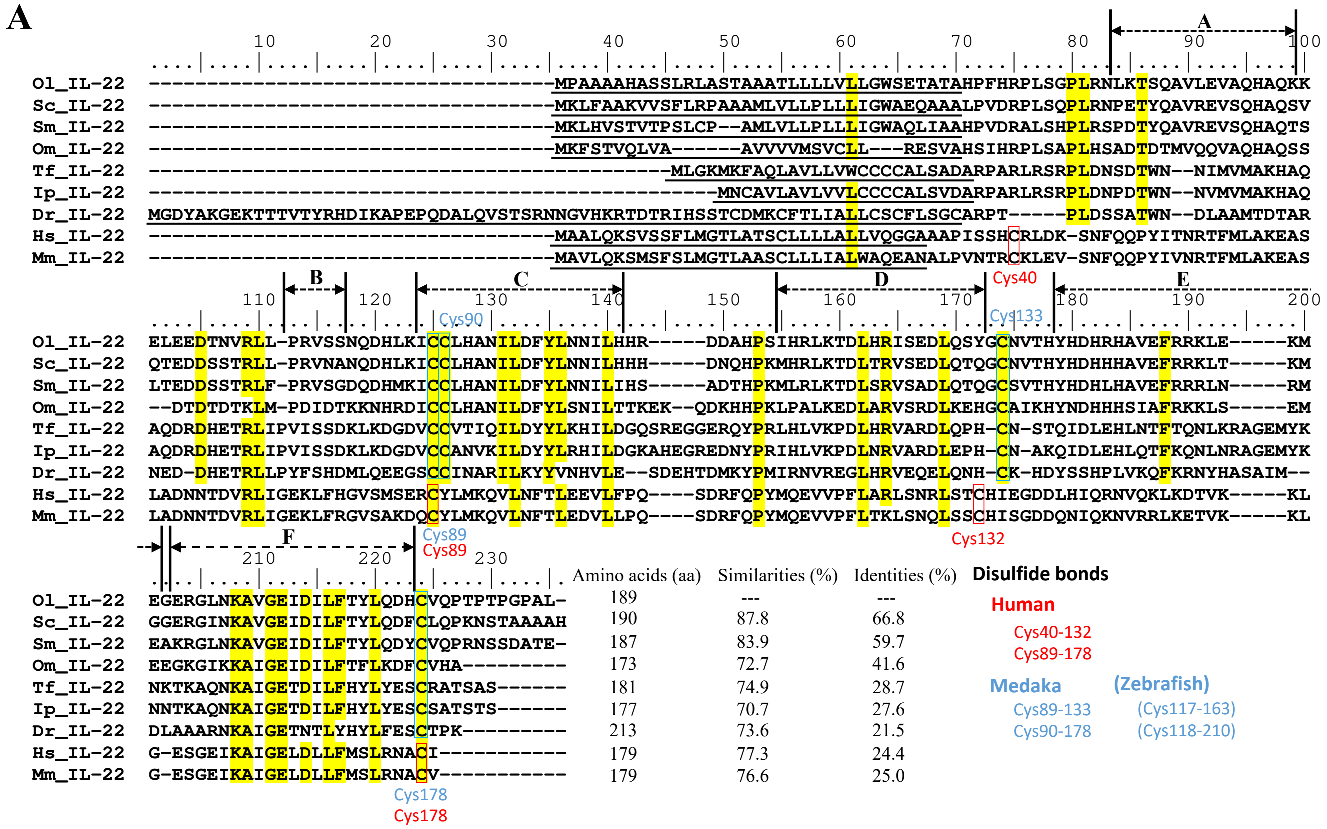

Fig. S1. Multiple alignments of the deduced protein sequences of IL-22 (**A**), IL-22RA1 (**B**), and IL-22BP (**C**) in Japanese medaka and other vertebrates. Identities and similarities of each IL-22, IL-22RA1, and IL-22BP protein with Japanese medaka IL-22, IL-22RA1, and IL-22BP peptide, indicated on the right side of the alignment. The amino acid residues highlighted in yellow represent matches of more than 75% among the aligned species. The letters (**A to F**) in the alignment of IL-22 show each range of alpha helix structures. Cysteine residues forming disulfide bonds are surrounded by red boxes (mammal) and blue boxes (teleost). In IL-22RA1 and IL-22BP, the ranges of functional domains (FNIII) and transmembrane regions predicted using SMART7 are shown by arrows. The species for the sequences used in these alignments are as follows; *Oryzias latipes* (Ol), *Siniperca chuatsi* (Sc), *Scophthalmus maximus* (Sm), *Oncorhnchus mykiss* (Om), *Tachysurus fulvidraco* (Tf), *Ictalurus punctatus* (Ip), *Danio rerio* (Dr), *Takifugu rubripes* (Tr), *Cynoglossus joyneri* (Cj), *Salmo salar* (Ss), *Esox lucius* (El), *Astyanax mexicanus* (Am), *Tachysurus fulvidraco* (Tf), *Cyprinus carpio* (Cc), *Homo sapiens* (Hs), and *Mus musculus* (Mm). Full names, abbreviations, and GenBank accession numbers of the sequences shown in this alignment are listed in **Table S2.**

Fig. S1. B

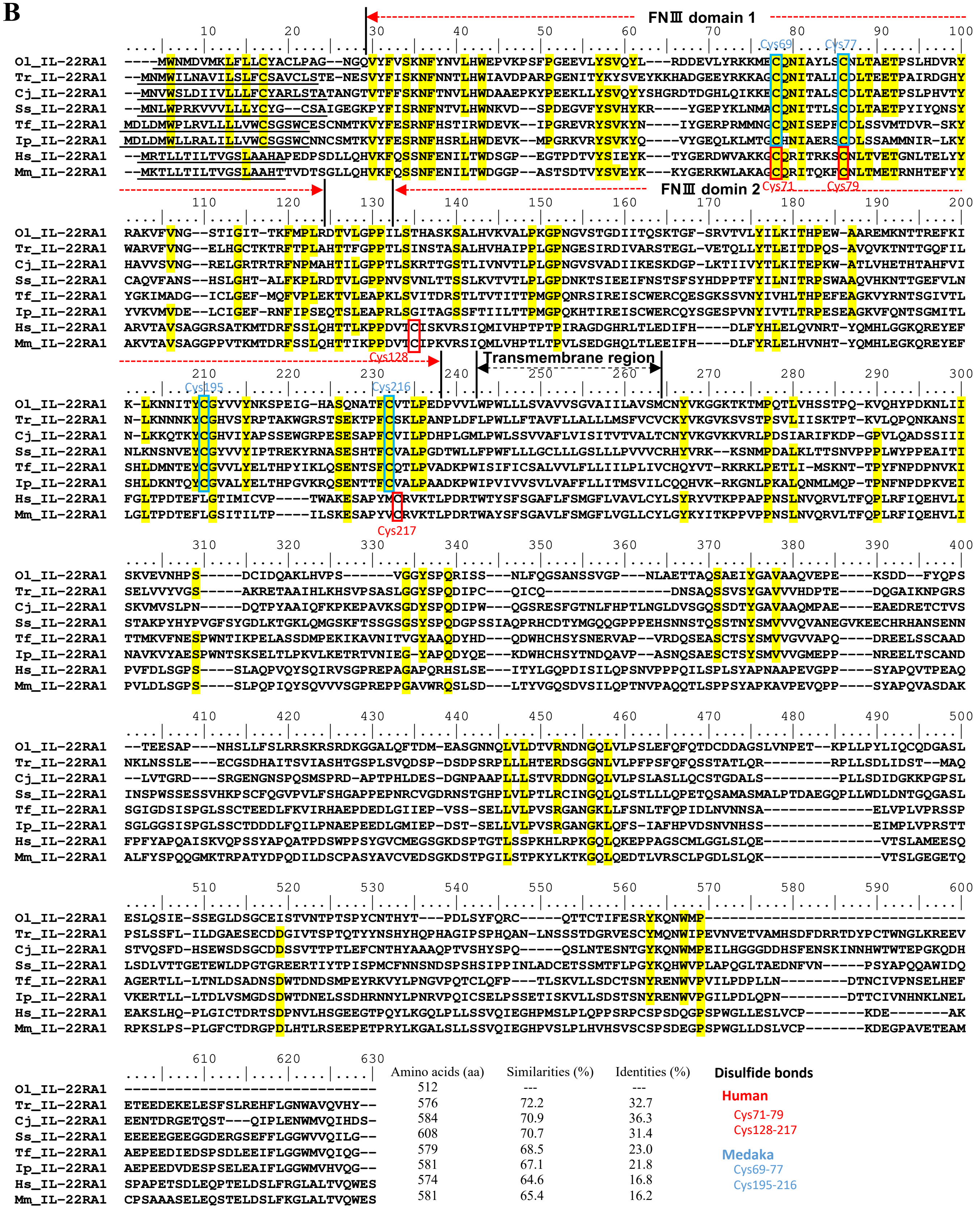

Fig. S1. Continued.

Fig. S1. C

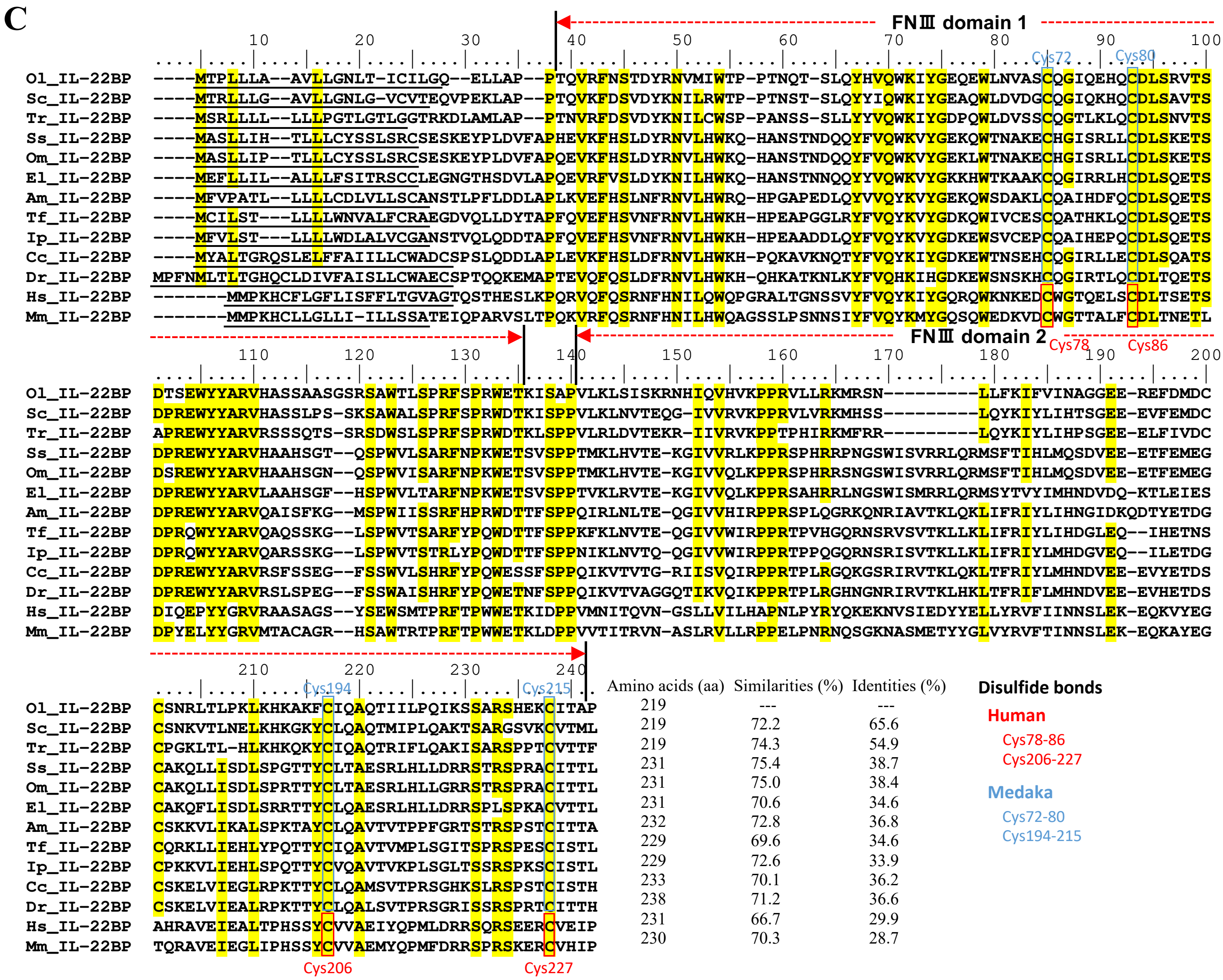

	Amino acids (aa)	Similarities (%)	Identities (%)
Ol_IL-22BP	219	---	---
Sc_IL-22BP	219	72.2	65.6
Tr_IL-22BP	219	74.3	54.9
Ss_IL-22BP	231	75.4	38.7
Om_IL-22BP	231	75.0	38.4
El_IL-22BP	231	70.6	34.6
Am_IL-22BP	232	72.8	36.8
Tf_IL-22BP	229	69.6	34.6
Ip_IL-22BP	229	72.6	33.9
Cc_IL-22BP	233	70.1	36.2
Dr_IL-22BP	238	71.2	36.6
Hs_IL-22BP	231	66.7	29.9
Mm_IL-22BP	230	70.3	28.7

Fig. S1. Continued.

Fig. S2.

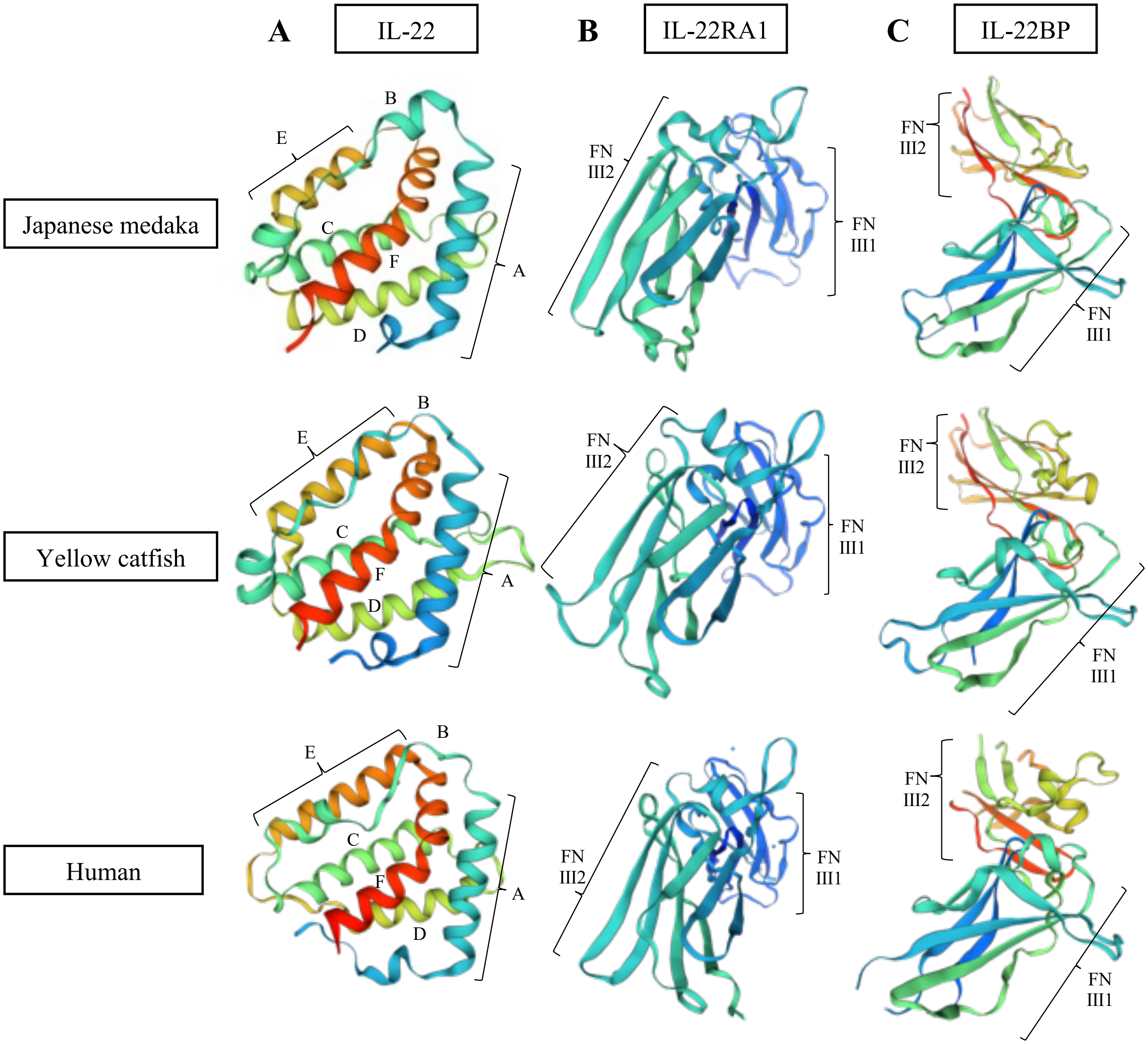

Fig. S2. 3D structures of IL-22 (**A**), IL-22RA1 (**B**), and IL-22BP (**C**) in human, yellow catfish, and medaka predicted using SWISS-MODEL. In the predicted 3D structures, the alpha helices A to F in IL-22 and the FNIII domain in IL-22RA1 and IL-22BP are shown. GenBank accession numbers of all nucleotide sequences are listed in **Table S2**.

Fig. S3.

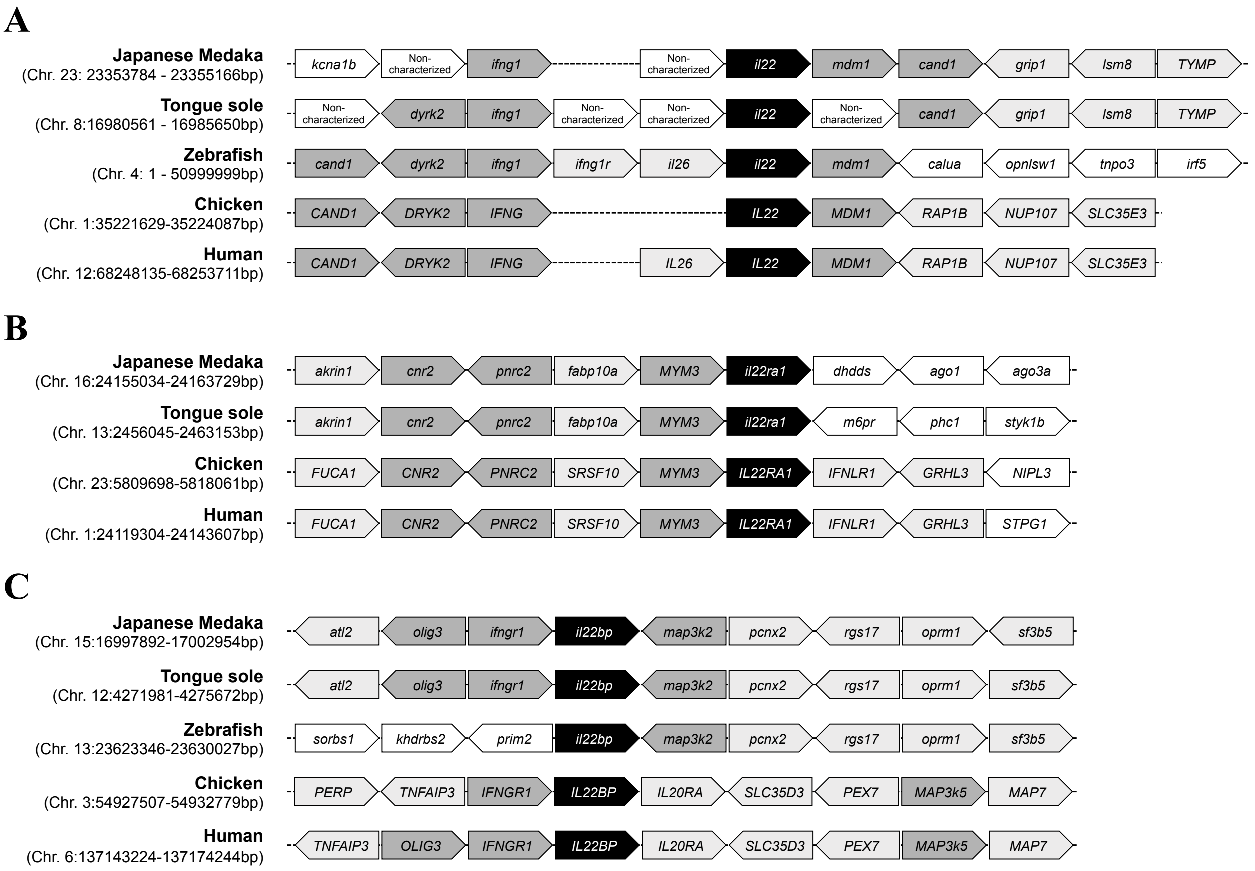

Fig. S3. Schematic representation of synteny for *il22* (**A**), *il22ra1* (**B**), and *il22bp* (**C**) in various species. *il22*, *il22ra1*, and *il22bp* highlighted by the black boxes. The dark gray and light gray boxes represent high and low gene homology, respectively.

Fig. S4.

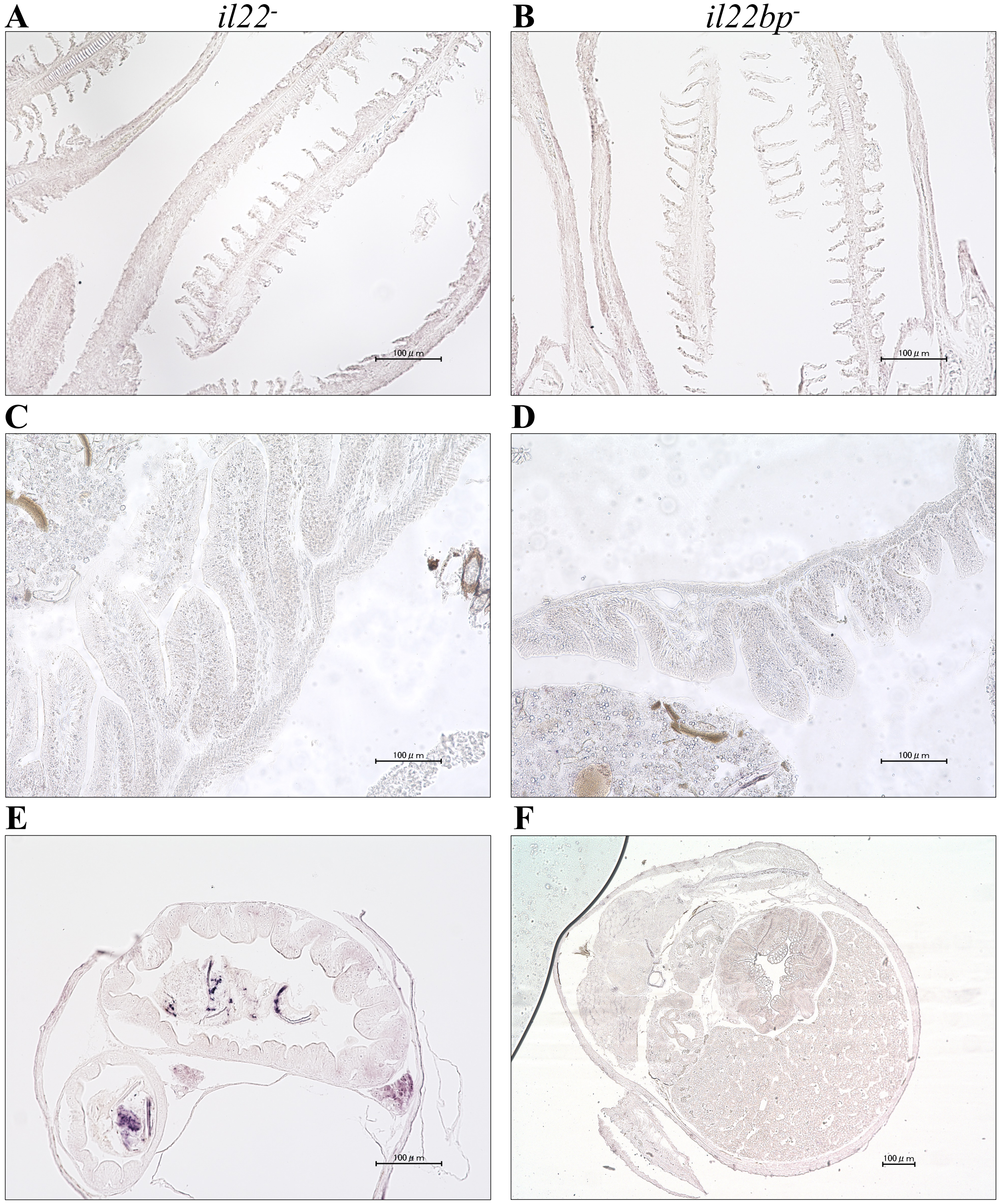

Fig. S4. Negative control (sense probe) of *in situ* hybridization (ISH) in medaka *il22* and *il22bp* mRNA localization. *il22* (**A**) and *il22bp* (**B**) expression in adult medaka gill; *il22* (**C**) *and il22bp* (**D**) expression in adult medaka intestine; *il22* (**E**) and *il22bp* (**F**) expression in larval medaka intestine. Scale bar: 100 μm. After 4% (v/v) paraformaldehyde fixation, the gills and intestines of healthy adult medaka and the whole body of the larvae were embedded in paraffin. DIG-labeled anti-sense RNA-probes were used for detection. After hybridization, color development was performed using AP-labeled anti-DIG IgG (sheep) and NBT/BCIP solution.

Fig. S5.

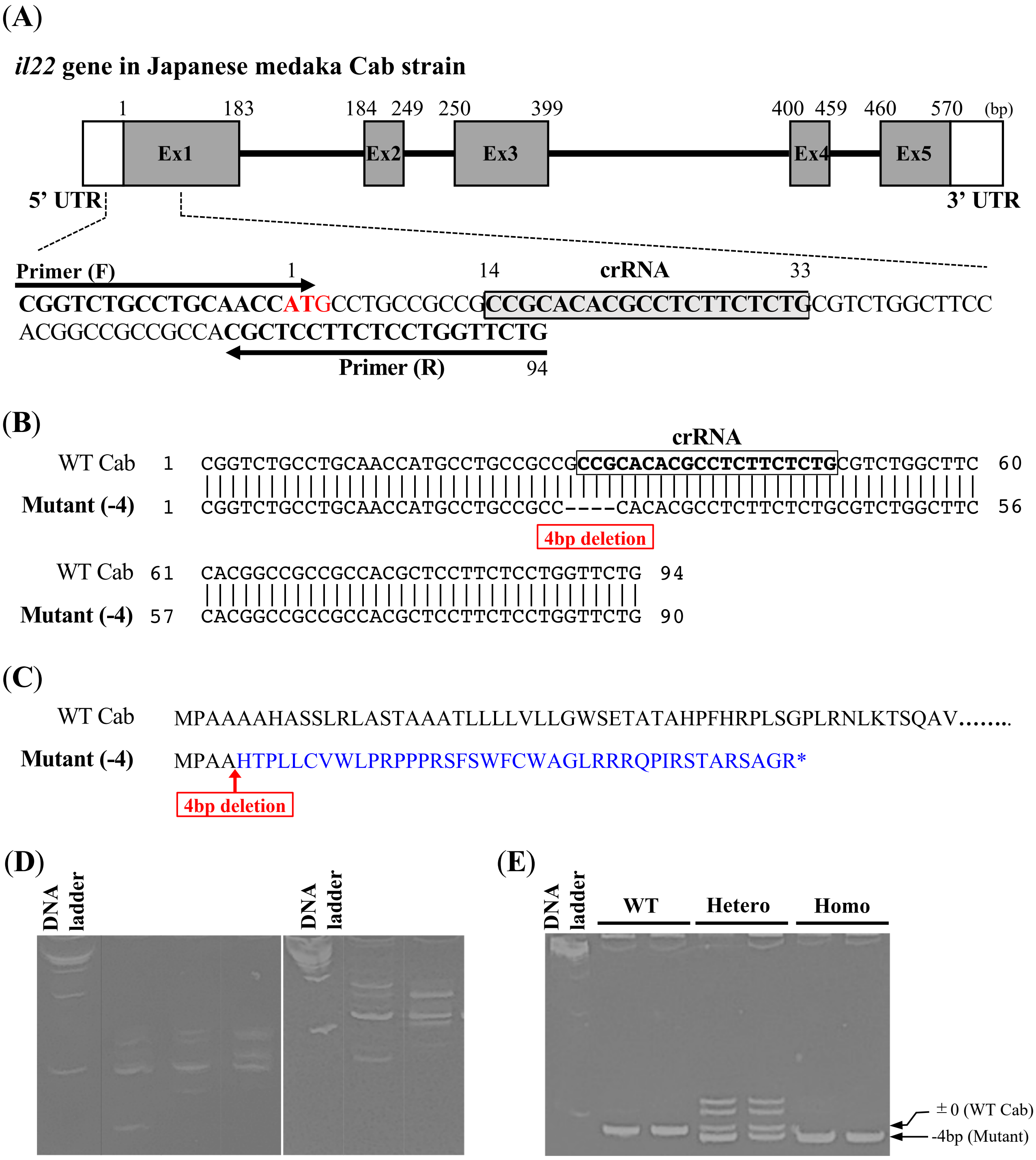

Fig. S5. crRNA regions, mutated sequences, and mutation efficiency of medaka *il22*. (**A**) crRNA regions in medaka *il22*. crRNA designed in exon 1 of *il22*. Mutated nucleotide (**B**) and amino acid sequences (**C**) in WT and KO strains. Mutated regions in filial generation zero (F0) founder and F3 homo individuals confirmed by sequencing. The asterisk (*) indicates the stop codon. Mutant strains contained premature stop codons in the amino acid sequences introduced by frameshift mutations. (**D**) Efficiency of mutation determined using crRNA and the heteroduplex mobility assay (HMA). (**E**) HMA for mutated medaka; a 4 bp deletion shown in a 12% agarose gel.

Fig. S6.

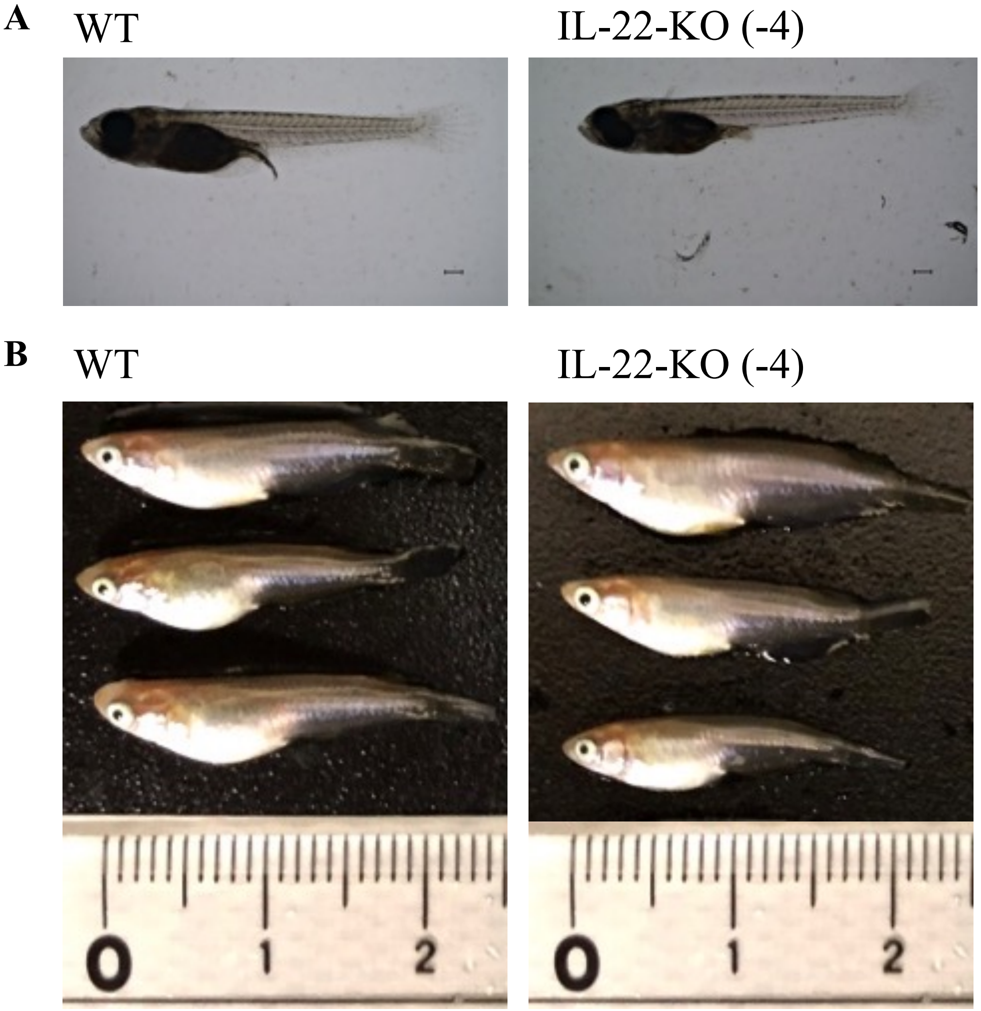

Fig. S6. Morphological comparison of WT and IL-22-KO medaka (-4). No morphological differences were observed between the KO strain (4 bp deletion strain) and WT medaka when 14 days post-fertilization larvae (**A**) and 3-month adult medaka (**B**) were observed.

Fig. S7.

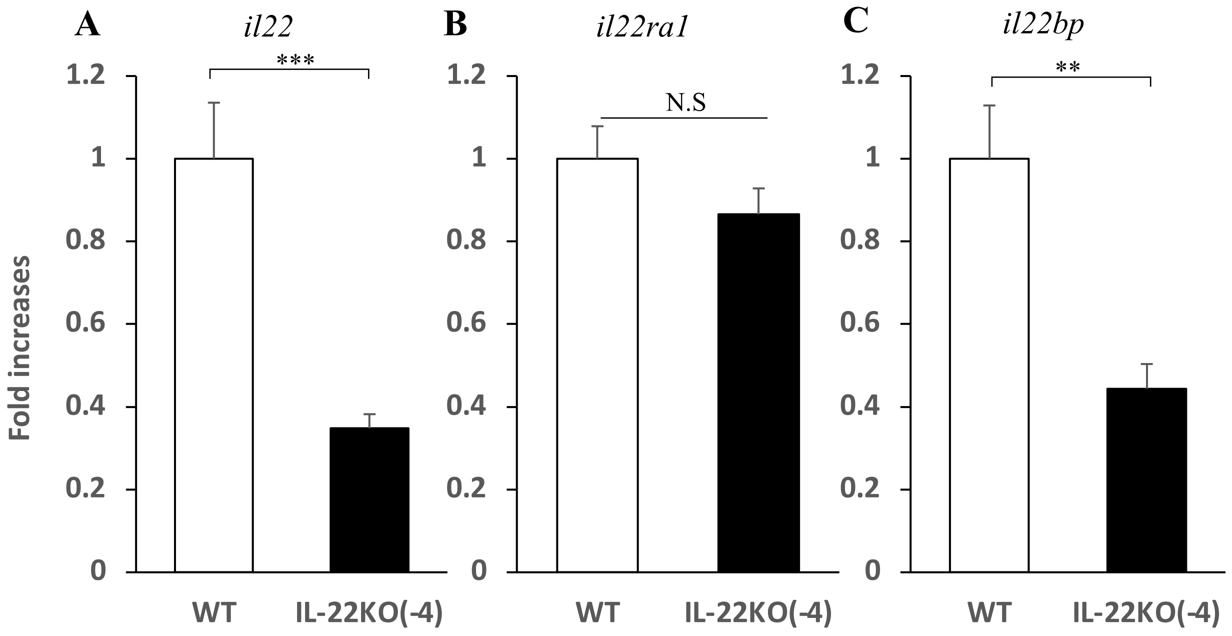

Fig. S7. Gene expression analyses of *il22* (**A**), *il22ra1* (**B**), and *il22bp* (**C**) in WT and KO medaka. Significant *il22* and *il22bp* downregulation was observed in KO (4 bp-deficient) medaka larvae compared with that in WT. Comparisons were performed on healthy medaka. The expression scale shows relative values when the expression in the WT group was set to 1. ***; $P < 0.001$, **; $P < 0.01$, *; $P < 0.05$ (two-tailed Student's *t*-test). Data shown were obtained from a single experiment (n = 7).

Fig. S8.

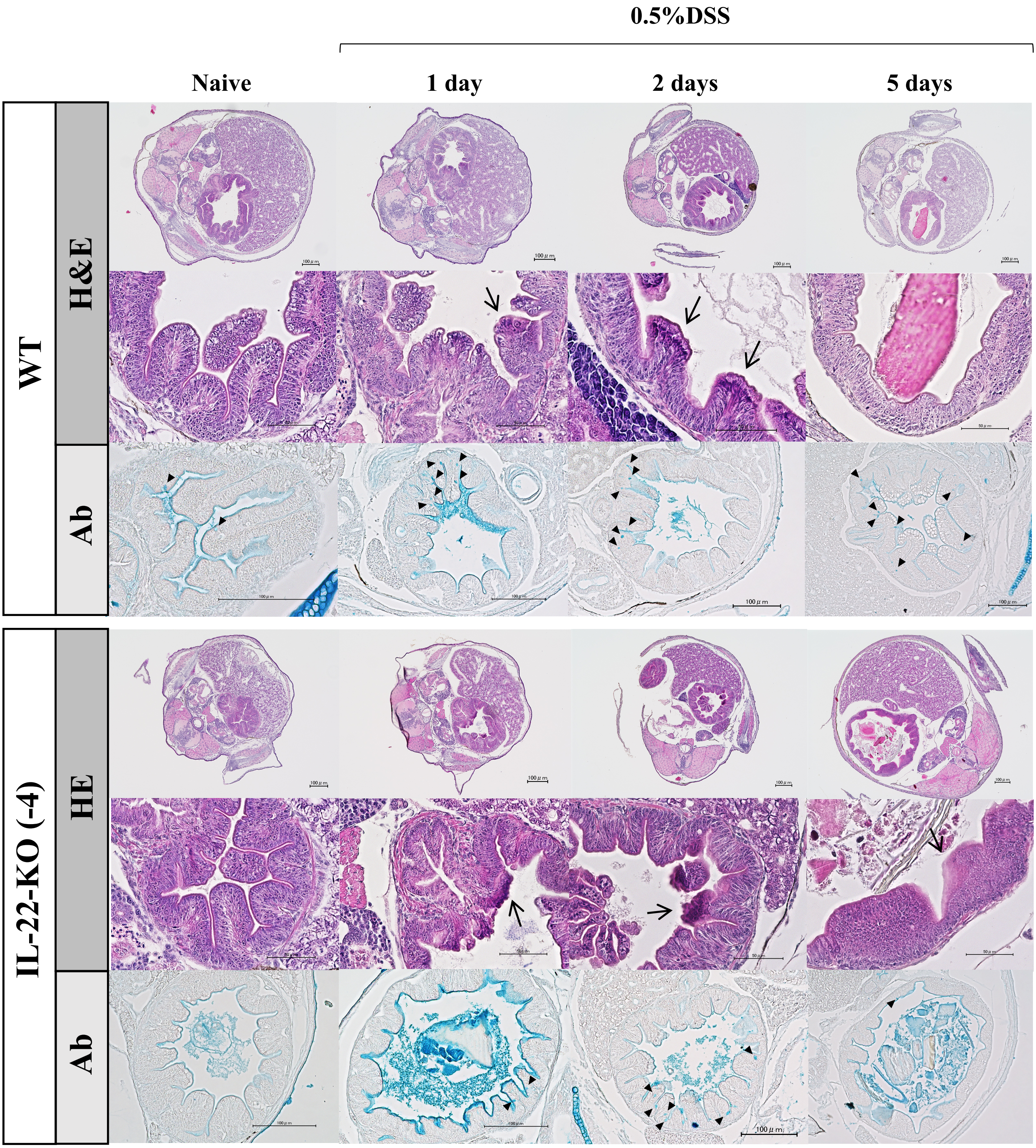

Fig. S8. Histological analysis of dextran sulfate sodium (DSS)-induced inflammation in WT and IL-22-KO medaka larvae. Samples of the anterior intestinal tissue sections from WT and IL-22-KO medaka at 1, 2, and 5 days after DSS stimulation and staining with hematoxylin-eosin (H&E) and Alcian blue (AB). All larval WT and IL-22KO medaka used were at 14 days post-fertilization. Arrows indicate the changes in tissue architecture caused by DSS. The arrowheads (▼) indicate the AB-positive cells.

Fig. S9.

A

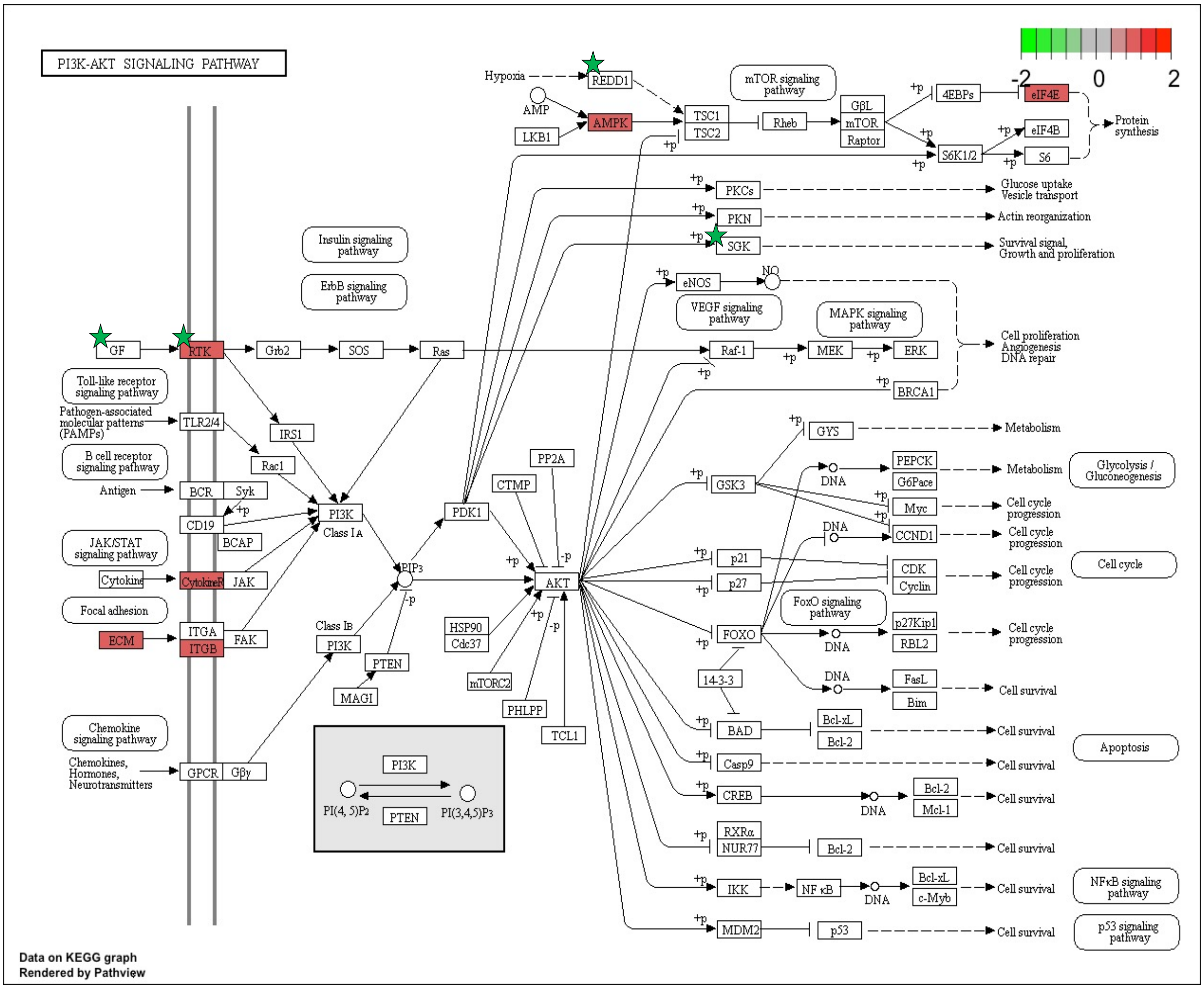

B

Gene_ID	Gene name	Gene Symbol	WT_DSS / WT*[1]		IL-22-KO (-4)_DSS / WT_DSS*[2]	
			Fold change	p_value	Fold change	p_value
**Up (WT / WT_DSS)**						
ENSORLG00000017636	eukaryotic translation initiation factor 4E	eif4e	1.974	0.039	1.071	n.s.
ENSORLG00000018568	Insulin receptor	insr	2.373	0.015	0.920	n.s.
ENSORLG00000003389	Integrin beta-3	itgb3	2.393	0.014	0.947	n.s.
ENSORLG00000022689	laminin subunit gamma-2	lamc2	2.005	0.042	0.938	n.s.
ENSORLG00000023149	oncostatin-M-specific receptor subunit beta	osmr	2.054	0.046	0.638	n.s.
ENSORLG00000001742	5'-AMP-activated protein kinase catalytic subunit alpha-2	prkaa2	2.401	0.015	0.679	n.s.
**Down (WT_DSS / IL-22-KO_DSS)**						
ENSORLG00000009171	DNA damage inducible transcript 4	ddit4	0.664	n.s.	0.613	0.044
ENSORLG00000009764	DNA damage-inducible transcript 4-like protein	ddit4l	4.172	n.s.	0.484	0.018
ENSORLG00000000289	fibroblast growth factor 19	fgf19	0.756	n.s.	0.287	0.015
ENSORLG00000024957	insulin-like growth factor-binding protein 1	igfbp1	1.476	n.s.	0.558	0.029
ENSORLG00000009059	serine/threonine-protein kinase Sgk1	sgk1	1.372	n.s.	0.577	0.034

Fig. S9. The PI3K-Akt signaling pathway maps of differentially expression gene (DEG) hits determined using data from the Kyoto Encyclopedia of Genes and Genomes (KEGG) database. (**A**) WT medaka stimulated with DSS showed upregulation of genes related to the "PI3K-Akt" pathway (red). IL-22-KO medaka stimulated with DSS showed downregulation of genes related to the "PI3K-Akt" pathway (green-star). (**B**) Eleven DEGs showed significant localization in WT and IL-22-KO medaka stimulated with DSS.

Fig. S10.

A

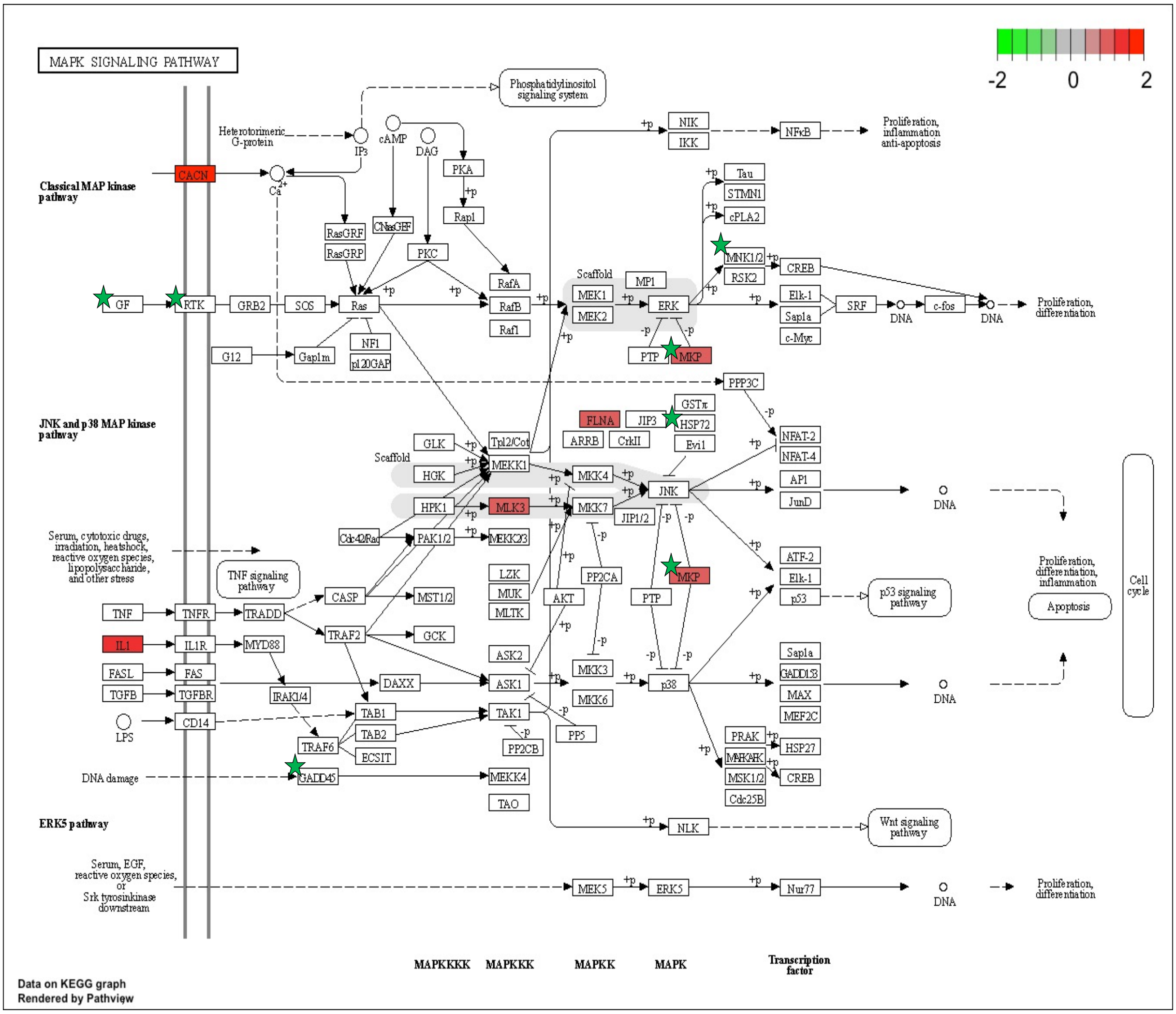

B

Gene_ID	Gene name	Gene Symbol	WT_DSS / WT*[1]		IL-22-KO (-4)_DSS / WT_DSS*[2]	
			Fold change	p_value	Fold change	p_value
**Up (WT / WT_DSS)**						
ENSORLG00000025805	voltage-dependent P/Q-type calcium channel subunit alpha-1A	cacna1a	2.523	0.020	0.889	n.s.
ENSORLG00000008618	voltage-dependent T-type calcium channel subunit alpha-1H	cacna1h	2.160	0.032	0.816	n.s.
ENSORLG00000015184	dual specificity phosphatase 5	dusp5	2.423	0.014	0.513	0.022
ENSORLG00000004247	filamin-C	flnc	1.921	0.041	0.868	n.s.
ENSORLG00000000217	interleukin-1 beta	il1b	2.545	0.012	1.056	n.s.
ENSORLG00000011136	mitogen-activated protein kinase kinase kinase 11	map3k11	2.040	0.047	0.974	n.s.
**Down (WT_DSS / IL-22-KO_DSS)**						
ENSORLG00000009024	MAPK interacting serine/threonine kinase 2	mknk2	1.041	n.s.	0.620	0.048
ENSORLG00000015184	dual specificity phosphatase 5	dusp5	2.423	0.014	0.513	0.022
ENSORLG00000000289	fibroblast growth factor 19	fgf19	0.756	n.s.	0.287	0.015
ENSORLG00000022536	growth arrest and DNA damage-inducible protein GADD45 beta	gadd45b	1.242	n.s.	0.593	0.047
ENSORLG00000030080	growth arrest and DNA damage-inducible protein GADD45 gamma	gadd45g	1.073	n.s.	0.545	0.026
ENSORLG00000006132	oryzias latipes heat shock protein 70 (hsp70-5), mRNA	hsp70-5	0.786	n.s.	0.476	0.038
ENSORLG00000023848	neurotrophic receptor tyrosine kinase 1	ntrk1	0.930	n.s.	0.527	0.026

Fig. S10. MAPK signaling pathway maps of differentially expressed gene (DEG) hits determined using data from the Kyoto Encyclopedia of Genes and Genomes (KEGG) database. (**A**) WT medaka stimulated with dextran sulfate sodium (DSS) showed upregulation of genes related to the "MAPK" pathway (red). IL-22-KO medaka stimulated with DSS showed downregulation of genes related to the "MAPK" pathway (green-star). (**B**) Twelve DEGs showed significant localization in WT and IL-22-KO medaka stimulated with DSS.

Fig. S11.

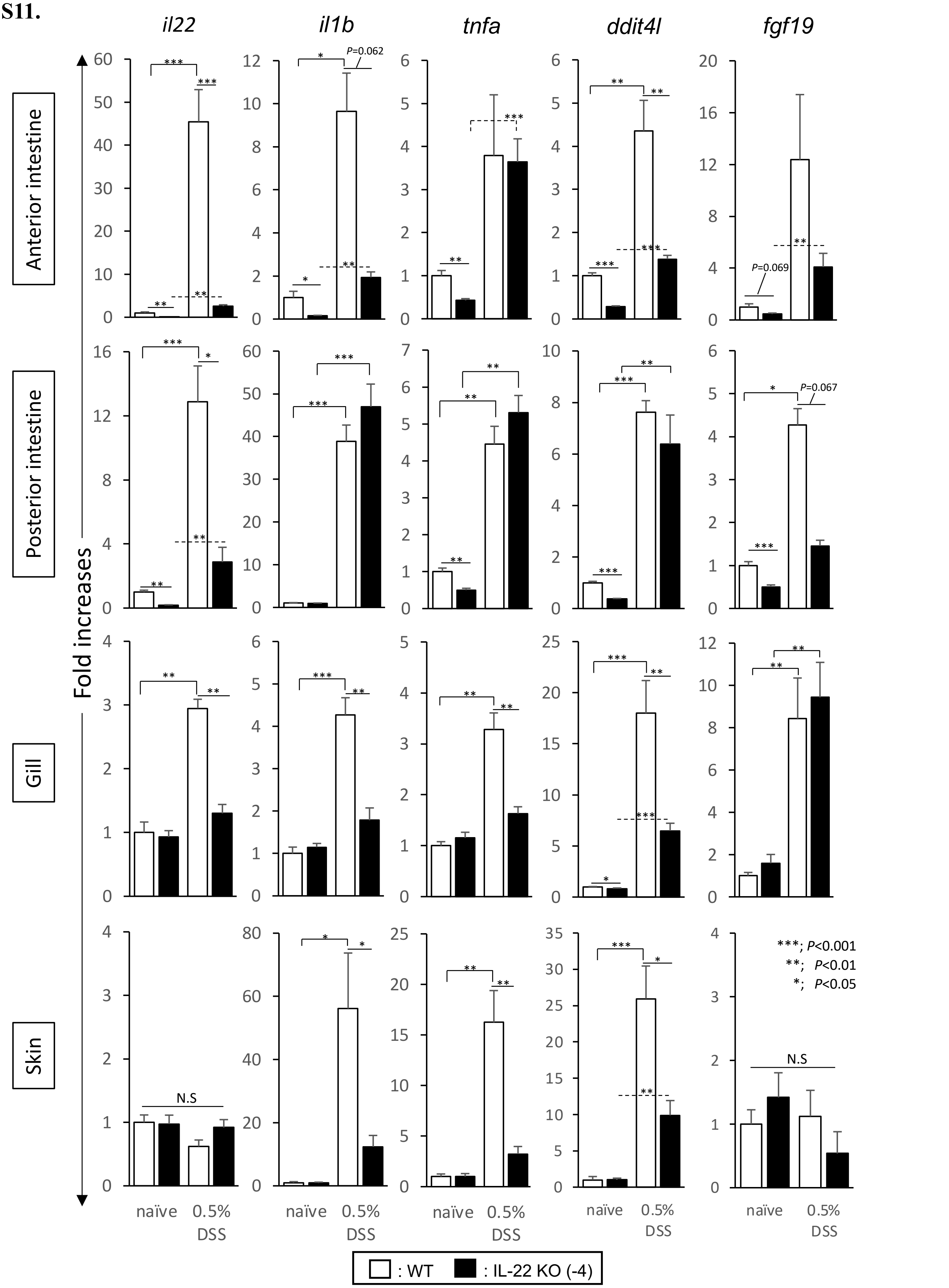

Fig. S11. Real-time PCR (qPCR) analysis of inflammatory cytokines and multiple genes related to the PI3K-Akt and MAPK signaling pathways. The expression of inflammatory cytokine (*il22*, *il1b*, and *tnfa*) and PI3K-Akt and MAPK signaling pathway-related genes (*ddit4l* and *fgf19*) in the intestines, gills, and skin of adult medaka was quantified using qPCR. *ddit4l* and *fgf19* were chosen as the target genes based on the qPCR and RNA-seq results in the dextran sulfate sodium (DSS) stimulation experiment using larval medaka. DSS stimulation was performed by the immersion method in the larval experiment. The expression scale shows the relative values obtained when the expression of the naïve WT group was set to 1. Data represent the mean ± SEM from five individual medaka in each group (n=5). ***; $P < 0.001$, **; $P < 0.01$, *; $P < 0.05$ (two-tailed Student's *t*-test).

Fig. S12.

A

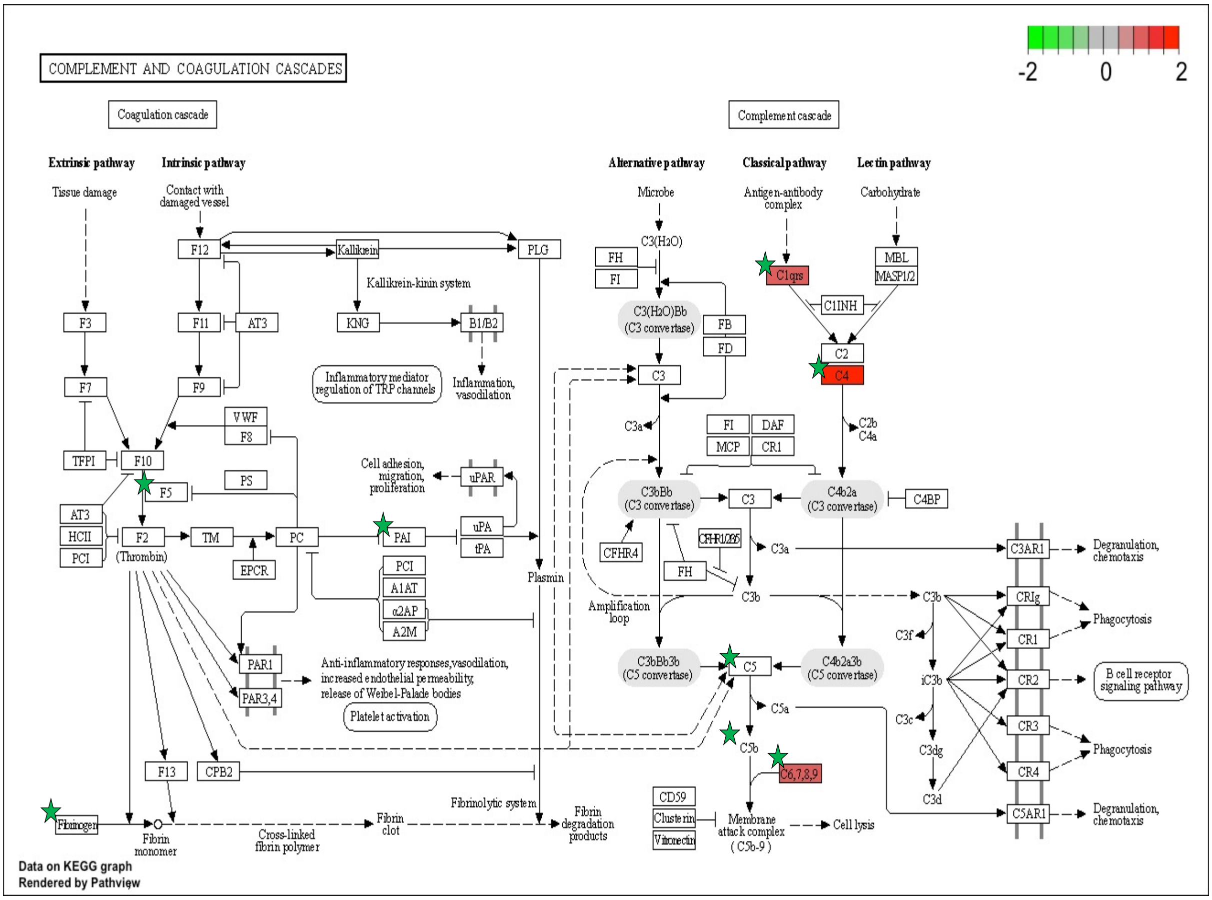

B

Gene_ID	Gene name	Gene Symbol	WT_DSS / WT*[1]		IL-22-KO (-4)_DSS / WT_DSS*[2]	
			Fold change	p_value	Fold change	p_value
**Up (WT / WT_DSS)**						
ENSORLG00000026388	complement C1q subcomponent subunit C	c1qc	2.361	0.014	0.148	n.s.
ENSORLG00000027110	complement C1q-like protein 2	c1ql2	3.038	0.035	0.367	0.038
ENSORLG00000006961	complement C4	c4b	5.141	0.005	0.303	0.019
ENSORLG00000014843	complement C6	c6	2.410	0.013	0.482	0.015
**Down (WT_DSS / IL-22-KO_DSS)**						
ENSORLG00000027110	complement C1q-like protein 2	c1ql2	3.038	0.035	0.367	0.038
ENSORLG00000012352	complement C1r	c1r	1.694	n.s.	0.482	0.038
ENSORLG00000006961	complement C4	c4b	5.141	0.005	0.303	0.019
ENSORLG00000006939	complement C4	c4b	1.383	n.s.	0.449	0.024
ENSORLG00000017552	complement C5	c5	1.484	n.s.	0.561	0.028
ENSORLG00000014843	complement C6	c6	2.410	0.013	0.482	0.015
ENSORLG00000014812	complement C7	c7	1.394	n.s.	0.594	0.042
ENSORLG00000013399	Coagulation Factor V	f5	1.671	n.s.	0.579	0.033
ENSORLG00000002453	fibrinogen alpha chain	fga	1.717	n.s.	0.614	0.042
ENSORLG00000008257	fibrinogen gamma chain	fgg	1.542	n.s.	0.626	0.047
ENSORLG00000010473	serpin family E member 1	serpine1	1.678	n.s.	0.488	0.038

C

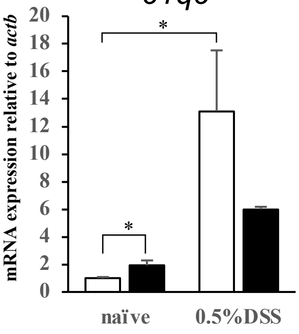

D

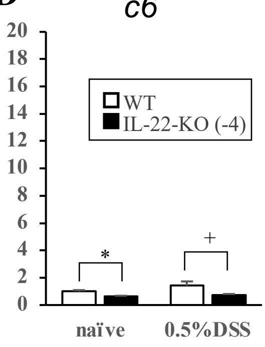

Fig. S12. Complement cascade maps of differentially expressed gene (DEG) hits determined using data from the Kyoto Encyclopedia of Genes and Genomes (KEGG) database. (**A**) WT medaka stimulated with dextran sulfate sodium (DSS) showed upregulation of genes related to the "complement" cascade (red). IL-22-KO medaka stimulated with DSS showed downregulation of genes related to the "complement" cascade (green-star). (**B**) Twelve DEGs showed significant localization in WT and IL-22-KO medaka stimulated with DSS. (C, D) Of the 12 DEGs, the expression of *c1qc* and *c6* was confirmed by qPCR. ***; $P < 0.001$, **; $P < 0.01$, *; $P < 0.05$, +; $P < 0.1$ (two-tailed Student's *t*-test). Data shown were obtained from a single experiment (n=7).

Fig. S13.

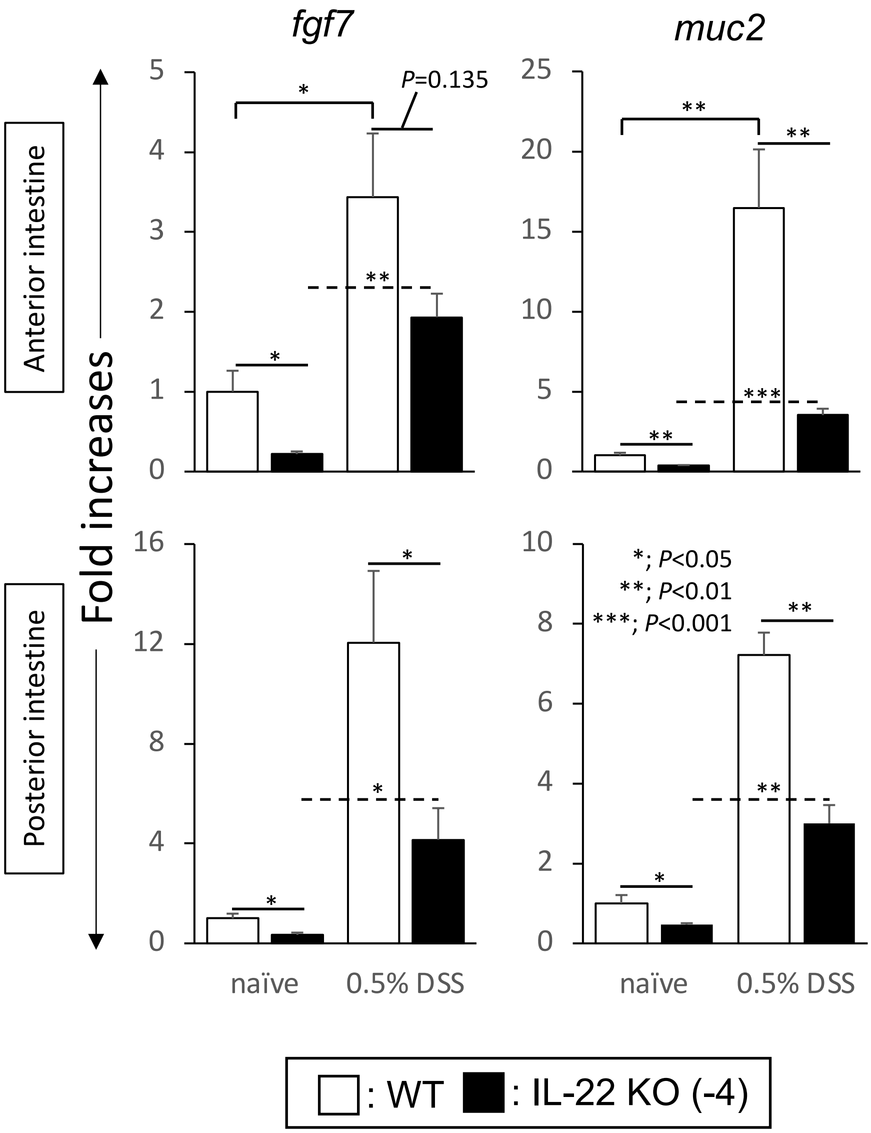

Fig. S13. Real-time PCR (qPCR) analysis of *fgf7* and *muc2* in the anterior and posterior intestines of adult medaka. Dextran sulfate sodium stimulation was performed by the immersion method in the larval experiment. The expression scale shows the relative values obtained when the expression of the naïve WT group was set to one. Data represent the mean ± SEM from five individual medaka in each group (n=5). ***; $P < 0.001$, **; $P < 0.01$, *; $P < 0.05$ (two-tailed Student's *t*-test).
